# Structural Characterization of the Full-Length Anti-CD20 Antibody Rituximab

**DOI:** 10.3389/fmolb.2022.823174

**Published:** 2022-04-11

**Authors:** Benny Danilo Belviso, Giuseppe Felice Mangiatordi, Domenico Alberga, Vincenzo Mangini, Benedetta Carrozzini, Rocco Caliandro

**Affiliations:** ^1^ Institute of Crystallography, CNR, Bari, Italy; ^2^ Cineca, Casalecchio di Reno, Italy

**Keywords:** structural comparison, anti-CD20 mab, molecular dynamics flexible fitting, small-angle X-ray scattering, full-length antibody

## Abstract

Rituximab, a murine–human chimera, is the first monoclonal antibody (mAb) developed as a therapeutic agent to target CD20 protein. Its Fab domain and its interaction with CD20 have been extensively studied and high-resolution atomic models obtained by X-ray diffraction or cryo-electron microscopy are available. However, the structure of the full-length antibody is still missing as the inherent protein flexibility hampers the formation of well-diffracting crystals and the reconstruction of 3D microscope images. The global structure of rituximab from its dilute solution is here elucidated by small-angle X-ray scattering (SAXS). The limited data resolution achievable by this technique has been compensated by intensive computational modelling that led to develop a new and effective procedure to characterize the average mAb conformation as well as that of the single domains. SAXS data indicated that rituximab adopts an asymmetric average conformation in solution, with a radius of gyration and a maximum linear dimension of 52 Å and 197 Å, respectively. The asymmetry is mainly due to an uneven arrangement of the two Fab units with respect to the central stem (the Fc domain) and reflects in a different conformation of the individual units. As a result, the Fab elbow angle, which is a crucial determinant for antigen recognition and binding, was found to be larger (169°) in the more distant Fab unit than that in the less distant one (143°). The whole flexibility of the antibody has been found to strongly depend on the relative inter-domain orientations, with one of the Fab arms playing a major role. The average structure and the amount of flexibility has been studied in the presence of different buffers and additives, and monitored at increasing temperature, up to the complete unfolding of the antibody. Overall, the structural characterization of rituximab can help in designing next-generation anti-CD20 antibodies and finding more efficient routes for rituximab production at industrial level.

## Introduction

Antibodies are multifunctional components of the immune system involved in cellular and humoral response. Most antibodies produced in response to self or foreign antigens are polyclonal, meaning they are produced by distinct type-B lymphocytes. As a result, they bind distinct antigen epitopes and have different specificity for the target. Instead, a monoclonal antibody (mAb) is generated from a specific type-B lymphocyte cell and recognizes with high affinity and specificity a unique epitope on a single antigen (Buss et al., 2012). mAbs are direct against a large number of antigens and have increasingly become important tools in biochemistry, molecular biology, and medicine, especially for the treatment of immunologic diseases or cancer (Buss et al., 2012).

Rituximab is the first mAb approved for the treatment of B-cell malignancies and lymphoma (Maloney et al., 1997). This antibody is effective in patients with relapsed or refractory CD20-positive follicular non-Hodgkin’s lymphoma and, despite it is widely recognized that it is not curative, it continues to be considered a benchmark for new generation mAbs (Marshall et al., 2017). Its target is the pan-B-cell marker CD20, a 35 kDa membrane protein ubiquitously expressed on circulating B cells (Casan et al., 2018), whose function and structure are not well defined, as to date, high-resolution structural information of this protein is not available. The protein could be involved in B-cell Ca^2+^ conductance (Bubien et al., 1993) or in calcium intracellular signaling (Walshe et al., 2008), and it could be formed by four antiparallel transmembrane helices and two conserved extracellular loops (ECL1 and ECL2) (Du et al., 2007). Moreover, there is no certainty about the CD20 functional organization. The prevailing hypothesis suggests that CD20 acts as a tetramer forming a plasma membrane ion channel (Klein et al., 2013) but, recently, a compact dimeric double-barrel assembly was proposed with no plausible ion permeation pathway (Rougè et al., 2020). CD20 is an ideal target for immunotherapy because: 1) it is present on the B-cells surface but not on that of stem cells or other normal tissues (Walshe et al., 2008); 2) it is expressed in over 95% of B-cell lymphomas; 3) it remains on the cell surface without substantial internalization after the antibody binding (Wrigley et al., 1983; Walshe et al., 2008).

To target CD20, the murine fragment antigen binding (Fab) region and a human fragment crystallizable (Fc) constant region (Reff et al., 1994) have been joint together to produce the chimera antibody today known as rituximab. The specificity of this protein drug for CD20 antigen resides in the murine regions, while the human part is required both for an effective therapeutic action and to decrease the immunogenicity of the protein drug (Grillo-López et al., 1999). Rituximab acts by depleting CD20^+^ cells *via* multiple mechanisms: antibody-dependent cellular toxicity, complement-dependent cytotoxicity, phagocytosis by macrophages and direct effect such as inhibition of cell proliferation, induction of apoptosis, and sensitization of cancer cells to chemotherapy (Wrigley et al., 1983). All of these mechanisms have been demonstrated independently *in vitro* (Yang et al., 2003) but it is still unclear which is the most important one *in vivo* and the way by which this anti-CD20 antibody targets each different pathway. Likewise, the molecular mechanism of CD20 recognition by rituximab is not clear yet. In this perspective, determining and characterizing the structure of the full-length antibody is essential to understanding how anti-CD20 therapy works at molecular level in the view of optimizing the therapeutic strategy, for instance in a combined administration with others chemotherapeutics. Unfortunately, the inherent molecular flexibility of the protein complicates its crystallization, a step that remains the primary bottleneck for the structure determination of the intact protein (Wrigley et al., 1983). While epitope mapping analysis and structural determination of the Fab region were achieved, crystallographic characterization and high-resolution structure of the intact mAb have not been reported, even if protocols to produce crystals are shown in the literature (Yang et al., 2003; Yang et al., 2019).

Mutagenesis experiments showed that the ^170^ANPS^173^ motif in the ECL2 extracellular loop of CD20 might be essential for the epitope recognition of rituximab (Du et al., 2008). Moreover, the Fab combining site consists of four complementarity determining regions forming a large and deep pocket to accommodate the epitope peptide (Du et al., 2007). Interestingly, this motif appears to be embedded into the pocket on the Fab domain surface and plays an essential role in the binding of rituximab (Du et al., 2007). Recently, Rougè and co-workers (2020) determined the structure of CD20 bound to rituximab Fab by using cryo-electron microscopy (cryo-EM),a structure that shows a single CD20 unit binds two Fab domains that, due to the orientation, belongs very likely to two rituximab molecules. Moreover, they highlighted that in addition to the highly solvent-accessible region of the known peptide ECL2, the secondary epitope ECL1 seems to be mainly recognized by the residues on the light-chain of the antibody, contributing substantially to the affinity of the antibody for CD20 (Grillo-López et al., 1999). Intermolecular Fab-Fab interactions seem to be facilitated by proximity between the primary epitopes of each CD20 molecule, and appear to further strengthening the tetrameric structure of the whole complex. Nevertheless, in the absence of the structure of full-length antibody, it is difficult to determine the detailed molecular mechanism of the CD20 recognition by rituximab and, therefore, its therapeutic mechanism of action.

Small-Angle X-ray Scattering (SAXS) allows investigating the average conformation of the macromolecular objects in solution, supplying structural information at low resolution (Blanchet and Svergun, 2013). The technique has been here applied to revealing the rituximab structural determinants, and studying its conformation in the presence of additives and by varying the temperature.

In this study, the information deficit of the SAXS data, due to the intrinsic low resolution of this technique, is compensated by effective modelling based on a combined use of a priori information and up-to-date computational protocols.

## Materials and Methods

### Sample Preparation

Anti-C20 having the same sequence of the active ingredient of rituximab (DrugBank accession number DB00073) was supplied by Fujifilm Diosynth Biotechnologies in two buffer solutions: the first one contained sodium citrate 35 mM, NaCl 150 mM at pH 6.5 (hereafter named buffer A), and the second one contained Tris 10 mM, NaCl 100 mM at pH 8.0 (hereafter named buffer B). Additives were bought from Sigma Aldrich. Buffer A and B were selected for their importance in the drug formulation and stabilization capability of rituximab. Indeed, sodium citrate is found in the list of excipients of rituximab provided by several pharma companies, such as MabThera. Regarding TRIS-HCl, rituximab crystallization has been reported in this buffer (Yang et al., 2003), suggesting that the antibody is quite stable in this condition.

### SAXS Data Collections

Data collections were carried out during two beamline sessions at ESRF, beamline BM29, and two beamline sessions at Diamond Light Source, beamline B21. Samples at mAb concentration ranging from 1 to 10 mg/ml were picked up from batch plates stored at 4°C and processed in flow mode, to reduce radiation damage. 28 frames were acquired for each concentration, with acquisition time of 1 s/frame. Buffer measurements before and after each sample were acquired for background subtraction. In a dedicated experiment, the antibody was gradually heated from 4°C to 85°C. For selected temperatures in this range, the sample was equilibrated for 5 min and SAXS data were collected to monitor the effect of heating on the structural stability of rituximab.

### SAXS Data Analysis

Raw SAXS 2-D images were processed by DAWN (Filik et al., 2017) to produce normalized and radially integrated SAXS curves. Averaging and background subtraction were performed by using PRIMUS (Konarev et al., 2003). For each sample, SAXS profiles measured at 1, 2, 4, and 6 mg/ml were manually merged in this order by using overlapping ranges of the momentum transfer (*q*), to generate a unique profile with improved statistics at high scattering angles and attenuated effect of particle–particle interactions at low scattering angles (Glatter and Kratky, 1982). PRIMUS was also used to perform the Guinier analysis and to determine the radius of gyration (*R*_*g*_) and the minimum *q* value (*q*_min_) to be used to infer size and shape information. The estimation for the related maximum *q* value (*q*_max_) was obtained by FIND_Dmax tool included in SCÅTTER (Förster et al., 2010). The pair distance distribution function *P(r)* of each dataset was determined by using GNOM (Semenyuk and Svergun, 1991) for *q* values between *q*_min_ and *q*_max_.

Raw data from different samples were compared by using Principal Component Analysis (PCA) and hierarchical clustering implemented in the program RootProf (Caliandro and Belviso, 2014). The data matrix formed by profiles composed by the logarithm of SAXS intensity as a function of *q* from several datasets was processed on site by this fast analysis tool, to obtain prompt information during the execution of the experiments, and then reprocessed off site, to investigate how the different experimental conditions of the sample influence the SAXS signal.

The following *ab initio* modeling procedure was performed to determine the molecular envelope from the *P(r)* function: 20 models were generated by using the program DAMMIF (Franke and Svergun, 2009). Modeling was performed by using the annealing procedure in slow mode to generate more accurate models. Models were then grouped according to their normalized spatial discrepancy (NSD) (Kozin and Svergun, 2001) by DAMCLUST (Petoukhov et al., 2012) and models belonging to the same cluster were averaged by DAMAVER (Volkov and Svergun, 2003). Resulting models were used as starting model to generate a final refined model by DAMMIN (Svergun, 1999). The ambiguity of shape reconstruction from the scattering profile has been assessed by the program AMBIMETER (Petoukhov and Svergun, 2015).

Rigid body fitting was performed by SASREF (Petoukhov and Svergun, 2005) by using the mAb model determined by homology modeling (*Homology Modelling*). Several conditions were tested: *i*) initial model constituted by a single domain containing the full-length mAb or by three separate domains: Fc and the two Fab arms; *2*) domains treated rigid or made flexible by normal mode analysis. The strategy that use the three separate flexible domains produced the best agreement with data. The program CRYSOL (Svergun et al., 1995) was used to compare atomistic models with SAXS profiles, and the program EOM (Tria et al., 2015) was used to apply the ensemble optimization method, which describes experimental SAXS data using an ensemble representation of atomic models. It was run by generating 20,000 initial models and by considering Fc and the two Fab arms as distinct rigid domains.

### Homology Modelling

The homology model of the antibody was built by using the X-ray structure of its Fab portion (pdb code: 4KAQ, Bzymek and Williams, 2014) and that of the intact human antibody IgG b12 (pdb code: 1HZH, Saphire et al., 2001) as template. More specifically, a consensus model based on both the selected templates, was built by following the Multi-template model type procedure available in PRIME (Schrödinger release 2018–4) and refined based on the energy-based protocol. The obtained homology model was pre-treated by means of the protein preparation module available from the SCHRÖDINGER SUITE [PROTEIN PREPARATION WIZARD: Sastry et al. (2013); EPIK: Greenwood et al. (2010), Shelley et al. (2007); PRIME: Jacobson et al. (2002), Jacobson et al. (2004)] which enabled us to: 1) add missing hydrogen atoms; 2) determine the optimal protonation and tautomerization states of the residues; 3) fix the orientation of any misoriented groups; 4) create disulphide bonds; 5) perform a final energy minimization. Notice that glycans present in the human antibody IgG b12 structure used as template were kept in the final homology model.

The ability of the followed homology model procedure to properly predict the antibody folding was challenged by using a machine learning (ML) algorithm named AlphaFold (Jumper et al., 2021), and in particular the web platform recently developed by Mirdita et al. (2022). Remarkably, a good matching was observed after comparing our homology model and the ML-based 3D structures returned by AlphaFold for the light chain (RMSD equal to 1.50 Å), the heavy chain of the Fab portion (residues 1–220 - RMSD equal to 4.10 Å) and that of the FC portion (residues 239–451–RMSD equal to 1.34 Å). A figure showing the superimposition between our homology model and that returned by AlphaFold is provided in the supporting information (Supplementary Figure S1).

### MD Simulations

The developed homology model was inserted in a periodic box extended by 13 Å in each direction from all protein atoms and filled with TIP3P water molecules (Jorgensen et al., 1983) by using the “solvate” plug-in of the Visual Molecular Dynamics (VMD) software suite (Humphrey et al., 1996). To neutralize system 18 Cl^−^ ions were added using the VMD’s “autoionize” plugin. The final system, consisting of 344917 atoms, was relaxed for 200 ps, applying harmonic restraints only to the protein atoms (force constant of 1 kcal/mol/Å). All MD simulations were performed using NAMD 2.10 (Phillips et al., 2005) and the CHARMM36 force field (Best et al., 2012). The full system was minimized and in order to remove steric clashes in the initial geometry, we applied a minimization and equilibration protocol consisting of four phases and reported in recent co-authored papers (Alberga et al., 2014; Alberga et al., 2017): *i*) minimization (2,500 steps) applying harmonic restraints (force constant *k* = 1 kcal mol^−1^ Å^−2^) on the protein atoms; *2*) equilibration at T = 310 K with protein atoms kept at fixed positions for 200 ps; 3) relaxing at T = 310 K for 200 ps by applying harmonic restraints only to the protein atoms (force constant *k* = 1 kcal mol^−1^ Å^−2^); 4) gradual heating from T = 10–310 K, increasing the temperature of 25 K every 40 ps. The SHAKE algorithm was employed to constrain all R–H bonds (Kräutler et al., 2001). Periodic boundary conditions were applied in all directions. A non-bonded cut-off of 12 Å was used, whereas the particle mesh Ewald (PME) (Darden et al., 1993) was employed to include the contributions of long-range interactions. All simulations were performed in an isothermal-isobaric ensemble (1 atm, 310 K) with a Nosè–Hoover Langevin barostat (Martyna et al., 1994; Feller et al., 1995) (oscillation period 200 fs, decay coefficient 100 fs) and a Langevin thermostat (Adelman and Doll, 1976) (damping coefficient 1 ps^−1^). The time step was set to 2 fs, and coordinates were saved every 10^5^ steps (200 ps). A MD trajectory of 300 ns was generated. The equilibration of the structure required less than 5 ns and thus the first 5 ns were removed from the analysis. All simulations were performed on the FERMI supercomputer at CINECA, Italy.

### SAXS Restrained Modelling

The steps of the modelling procedure applied to SAXS data are illustrated in Supplementary Figure S2. The protocol has been conceived to apply experimental restraints in both reciprocal and direct space to the atomistic models generated by MD. *Ab initio* biochemical knowledge, embedded into the homology model, is used as input for running unrestrained MD according to the protocol described in *MD simulations*. In parallel, the SAXS profile, representing experimental data in the reciprocal space, is used to perform the *ab initio* modelling of the molecular envelope, representing the experimental information projected in the direct space. Reciprocal space data are used to select the best model generated by free MD, i.e., the model whose calculated scattering profile shows the best fit (lowest χ^2^) with the observed SAXS profiles. This model was subjected to a further run of MD where direct space data have been used as restrain, in the form of the experimental molecular envelope. The best model is finally identified as that having the lowest χ^2^ among those produced by the restrained simulation.

The restrained MD has been performed by adapting the Molecular Dynamics Flexible Fitting (MDFF) tool (Trabuco et al., 2008), originally developed in VMD (Humphrey et al., 1996) to include in simulations electron density maps derived from X-ray diffraction or cryo-EM measurements. The MDFF potential is defined as $V\left(r\right)=k\left[1-\frac{\rho \left(r\right)}{\rho_{max}}\right]$ , where ρ(r) is the molecular envelope at the grid point r , $\rho_{max}$ is its maximum value, and k is scaling factor defining the strength of the molecular envelope restrain, which was set to 0.1. This atomic potential has been applied only to C_α_ atoms of the mAb. In order to avoid structural artifacts that could arise by applying the relatively large force generated by this potential, MDFF simulation has been carried out by applying secondary structure restraints and tools for detecting and prevent generation of *cis* peptide bonds and chirality errors. The initial model was treated in the same way as the unrestrained MD, i.e., it was inserted in a periodic box extended by 13 Å in each direction from all protein atoms, filled with TIP3P water molecules, and 18 Cl^−^ ions. A non-bonded cut-off of 16 Å was used, while contributions of long-range interactions were included by using the particle mesh Ewald (PME). All simulations were performed in an isothermal-isobaric ensemble (1 atm, 300 K) using the CHARMM36 force field (Best et al., 2012) and a Langevin thermostat (damping coefficient 5 ps^−1^). The time step was set to 1 fs and coordinates were saved every 1,000 steps (1 ps). A MD trajectory of 2 ns was generated.

### Comparison Among Models

The atomistic models of the full-length antibody were compared by calculating their geometric properties by using VMD scripts developed *ad hoc*. Given the asymmetry of rituximab, the farther and closer arms to Fc will be hereafter referred to as Fa and Fb, respectively. The center of mass of the whole antibody and of the Fc, Fa, Fb domains were determined and used to calculate a number of geometrical features, such as their mutual distances and angles. We also calculated angles between the inertia planes, the radius of gyration and the solvent accessibility surface area of individual domains. Geometrical features from different models were analyzed by using the PCA and models were clustered in the space of selected principal components. These analyses were performed by using procedures present in RootProf (Caliandro and Belviso, 2014). These tools were used to select the best set of geometrical features that conveniently describe the mAb models, and to classify the latter according to the selected features.

The elbow angle of the Fab, i.e., the angle between the pseudo-two-fold axes defined by aligning the light chain portion of the variable and constant domains onto the heavy chain one (Sotriffer et al., 1998; Stanfield et al., 2006), has been calculated by the program phenix.fab_elbow_angle included in the PHENIX package (Liebschner et al., 2019).

The Root Mean Square Deviation (RMSD) of C_α_ atoms was calculated by the program SUPERPOSE (Krissinel and Henrick, 2004).

## Results

### Static Structural Characterization

The normalized Kratky plot (Supplementary Figure S3) shows two peaks, the first of which is well apart the position expected for globular compact particles (Durand et al., 2010; Receveur-Brechot and Durand, 2012). This suggests a high flexibly and/or asymmetry of the molecule under investigation.

**FIGURE 1:**
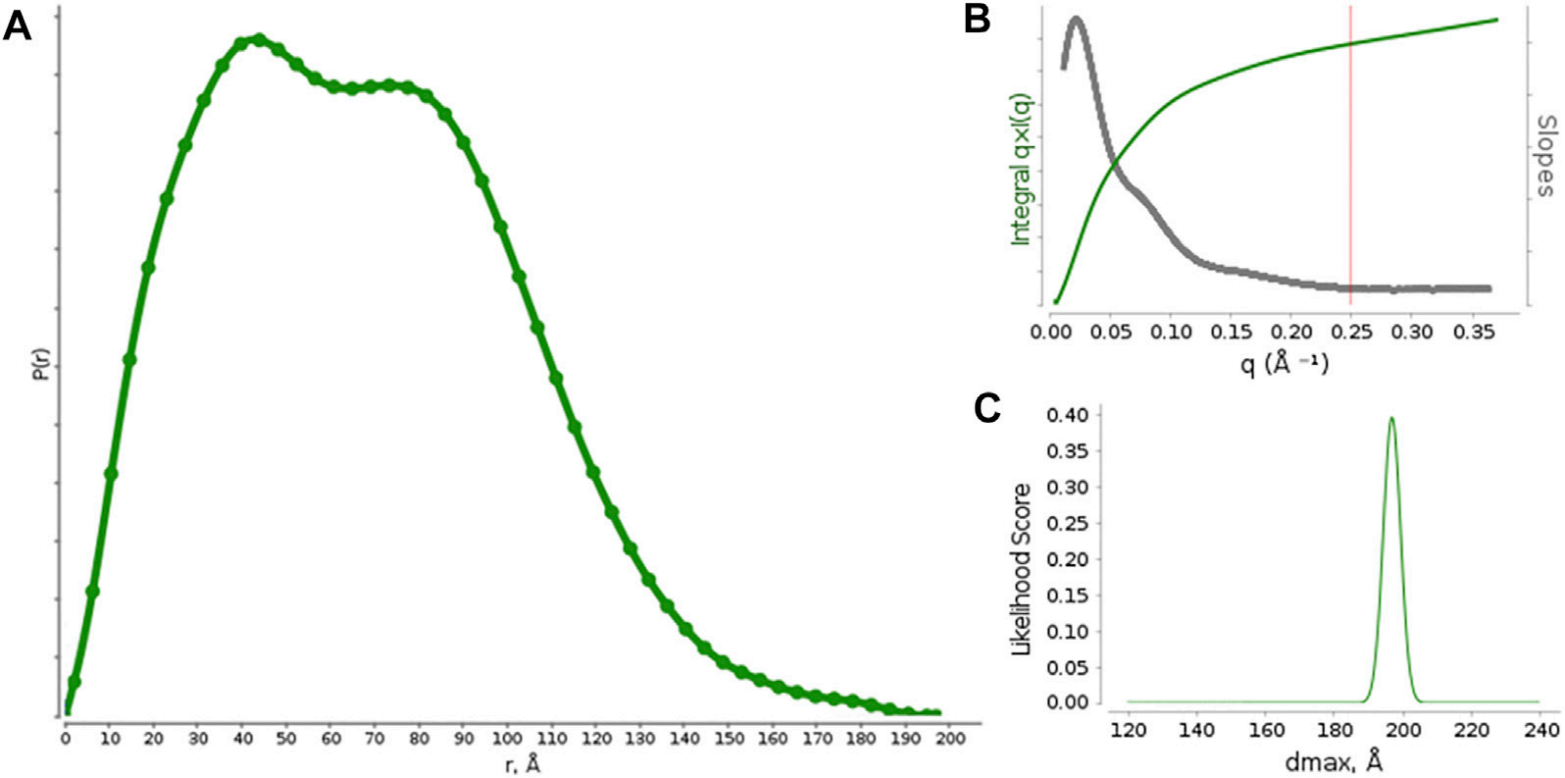
**(A)** Pair distribution function calculated from the measured SAXS profiles for rituximab. **(B)** graph with suggested *q*_max_ (red vertical line) based on the q * I (q) vs q curve and its derivative. **(C)** Likelihood score of *D*_max_ values calculated for different alpha values by using the Moore function to model *P(r)*.

Pair distribution function *P(r)* calculations (Figure 1) were optimized against different functions available in SCÅTTER. The best performance was obtained by the Moore method L1-Norm second derivative with constant background, which provided *R*_*g*_ = 52 Å and *D*_max_ = 197 Å. The optimal *q*_max_ was 0.25 Å^−1^ (Figure 1A) and the *D*_max_ likelihood score shows a single sharp peak (Figure 1B), suggesting a good model-data agreement and the absence of background subtraction issues. The data resolution was estimated to be 25.5 Å.

The *P(r)* function shows two maxima, occurring at *r* ∼ 40 Å and *r* ∼ 85 Å, which represent the most frequent interatomic distances within the predominant structure adopted by the antibody. According to previous reports on small-angle scattering analysis of antibodies, the first peak (∼40 Å) could results from the most commonly occurring distance within a single domain (Fab or Fc), by considering that each domain is approximately 8 nm long, and the second one (∼85 Å) could be related to the most common inter-domain distance within the whole structure of the antibody. (Boehm et al., 1999; Furtado et al., 2004). Moreover, the presence of two peaks in the *P(r)* function distinct is a clear sign that the antibody structure is quite rigid and shows the single Fab and Fc domains well apart each other.

The *P(r)* function has been used as input to calculate the molecular envelope, which required a computer-intensive procedure due to the high ambiguity of the scattering data. Indeed, the *P(r)* was found to be compatible with 809 shape categories, with an ambiguity score of 2.9 (scores values exceeding 2.5 points are considered very ambiguous for molecular envelope determination). 20 fast envelope determinations and the clustering procedure performed on such envelopes provides 3 clusters, formed by 10, 5 and 3 elements, with 2 isolated elements. The average envelopes from the two more populated clusters were further averaged to form the final envelope, which was used as experimental restrain to generate an atomistic model of the antibody.

**FIGURE 2:**
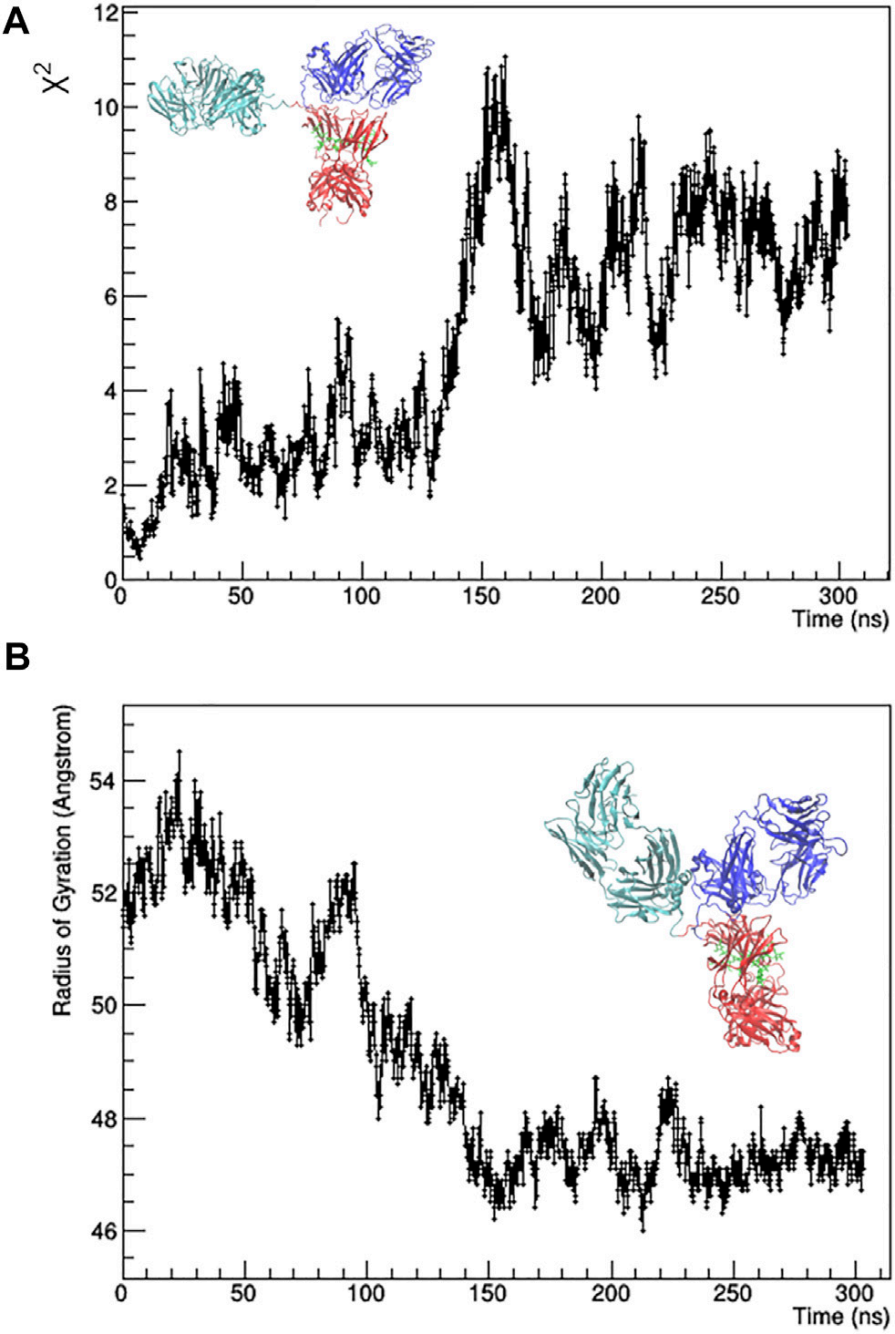
Results of the unrestrained molecular dynamics. χ^2^ value of the fit between calculated and observed SAXS profile **(A)** and radius of gyration **(B)** as a function of the simulation time. The best fitting model (on the left) and the final one (on the right) are shown. Fa, Fb and Fc domains are coloured in cyan, blue and red, respectively. Glycans are coloured in green and put in licorice representation.

The individual frames of the MD trajectory were checked against SAXS data, i.e., they were used for calculation with CRYSOL to fit the 1D-experimental curve with those calculated from the MD-simulated mAb models. The resulting χ^2^ values as a function of the simulation time (Figure 2A) shows that the best agreement between experimental data and MD-simulated models is reached after 7 ns, whilst after 150 ns the simulated model moves away from the experimental data. The main difference between the initial and the final configuration is an approach of the Fa domain to the center of the antibody, which determines a decrease of the *R*_*g*_ values (Figure 2B) and partially restores the symmetry around the Fc domain.

**FIGURE 3:**
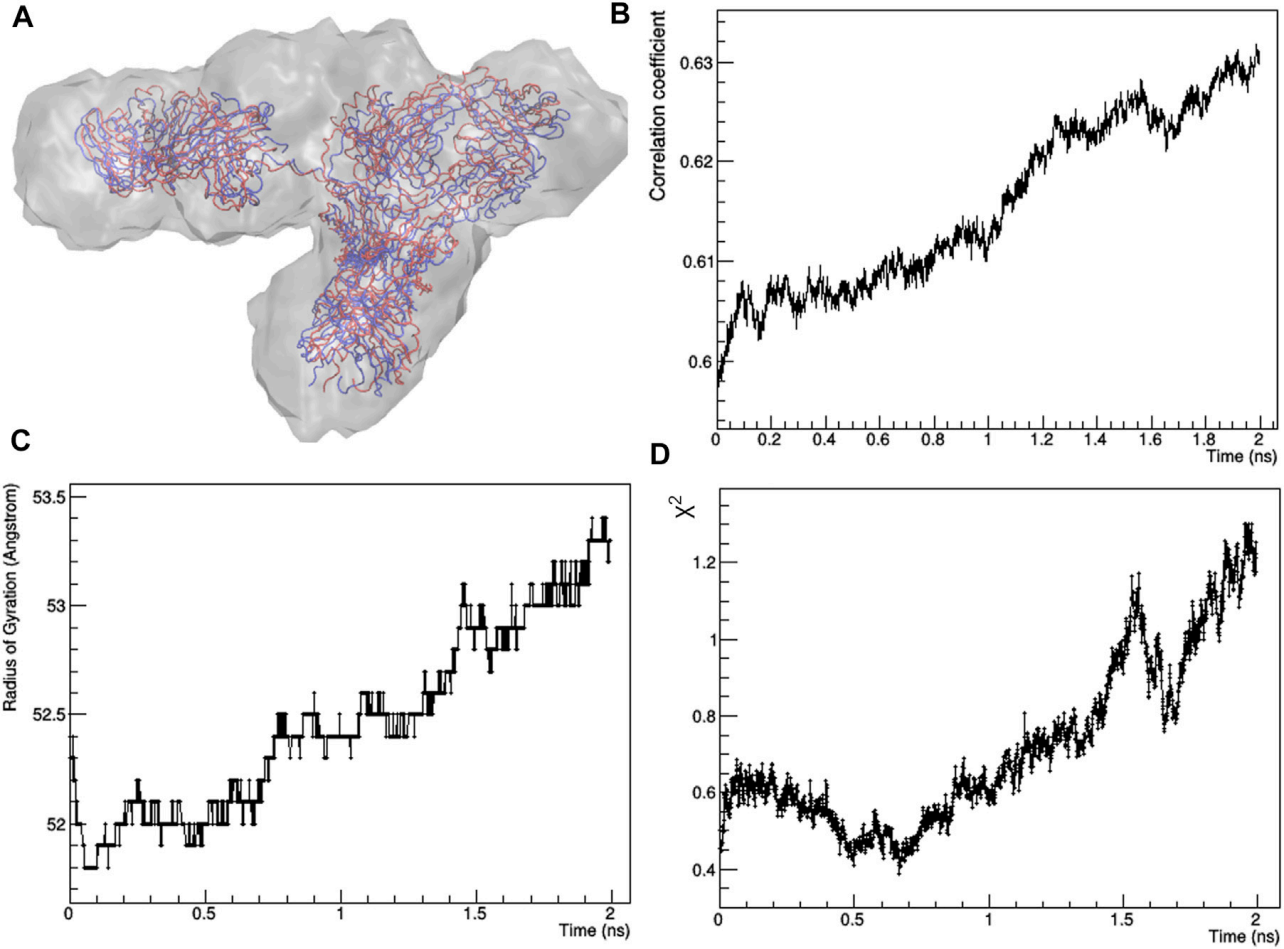
Results of the restrained molecular dynamics carried out by using the MDFF protocol: **(A)** starting (red) and final (blue) structural models superposed to the experimental molecular envelope; **(B)** Pearson’s correlation factor between calculated and observed molecular envelopes; **(C)** radius of gyration as a function of the simulation time; **(D)** χ^2^ value of the fit between calculated and observed SAXS profile as a function of the simulation time.

**FIGURE 4:**
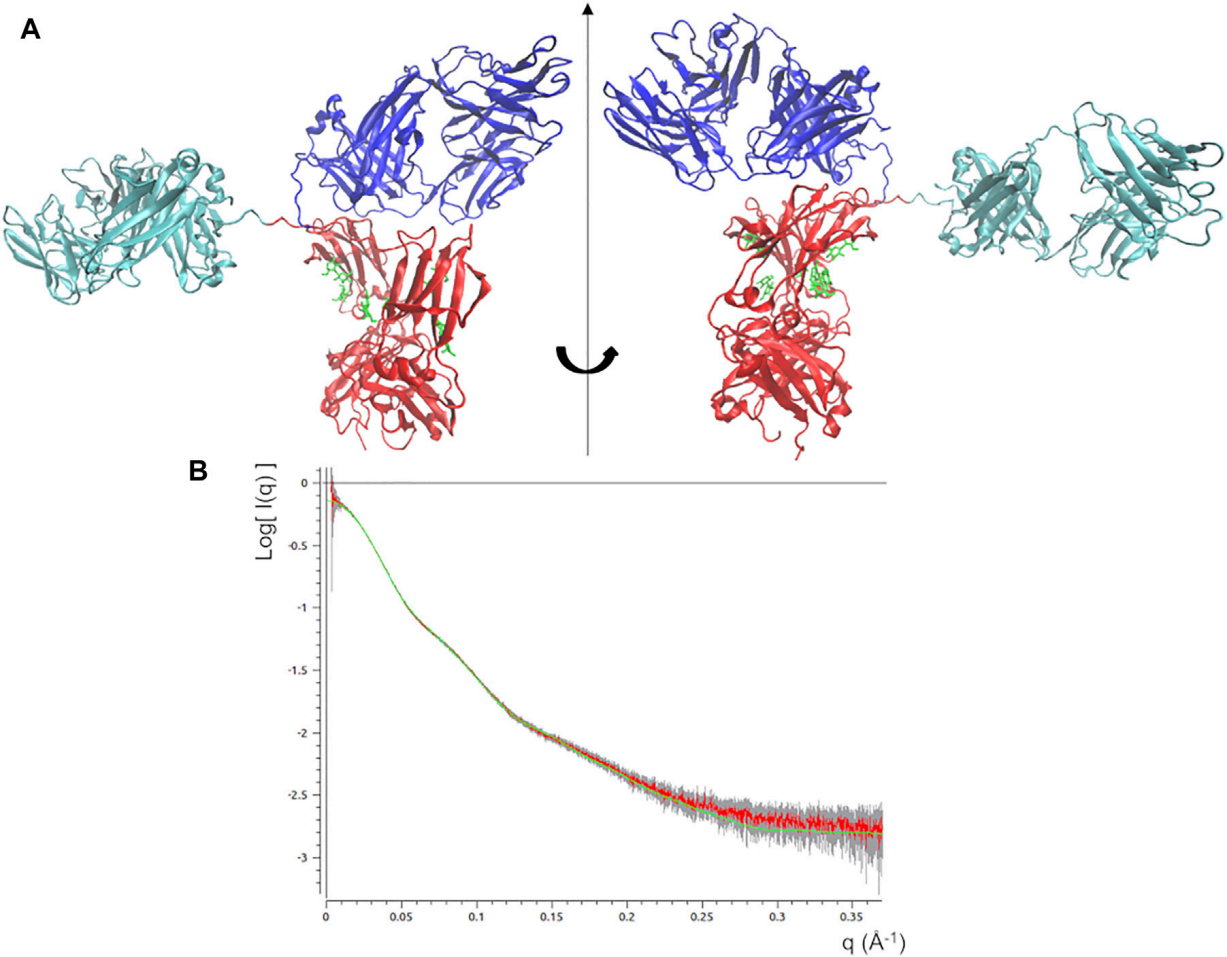
**(A)** Best fitting model obtained by the static modelling procedure based on the MDFF protocol. Fa, Fb and Fc domains are coloured in cyan, blue and red, respectively. Glycans are coloured in green and put in licorice representation. **(B)** Best fit of the observed SAXS profile (red line) along with experimental errors (grey bar) and that calculated scattering profile from the above model (green line).

The best fitting model (χ^2^ = 0.44) was taken as input for a local optimization procedure, which has been implemented according to the MDFF approach. MDFF results show that the procedure is able to induce conformational changes that lead the mAb atomistic model to perfectly fit the experimental molecular envelope (Figures 3A,B), as a result of a slight increase in the *R*_*g*_ values (Figure 3C). However, *χ* ^*2*^ decreases only in the first 0.7 ns, pointing out that the scattering calculated from the remaining conformations of the MD trajectory diverges to that of the experimental SAXS profile (Figure 3D). This can be explained as a mismatch between the restraint imposed in the direct space (the molecular envelope) and the agreement with data in the reciprocal space, which could be due to the previously mentioned ambiguity in the *ab initio* generation of the molecular envelope. As a trade-off between data agreement in direct and reciprocal space, the molecular model having the lowest χ^2^ value when fitted with the SAXS profile (χ^2^ = 0.39, reached after 0.67 ns of simulation time) was selected among those visited during the MDFF trajectory. This model, shown in Figure 4A, has a better agreement with SAXS data compared to the models generated by the unrestrained MD (Figure 4B) and represents our best model of the rituximab structure in solution. It has been deposited in the SASBDB database (Valentini et al., 2015), under the entry SASDMX3 (https://www.sasbdb.org/data/SASDMX3). It is worth noting that an unrestrained MD initiated from the same input model of the MDFF simulation did not produce any model in better agreement with experimental data (data not shown).

**FIGURE 5:**
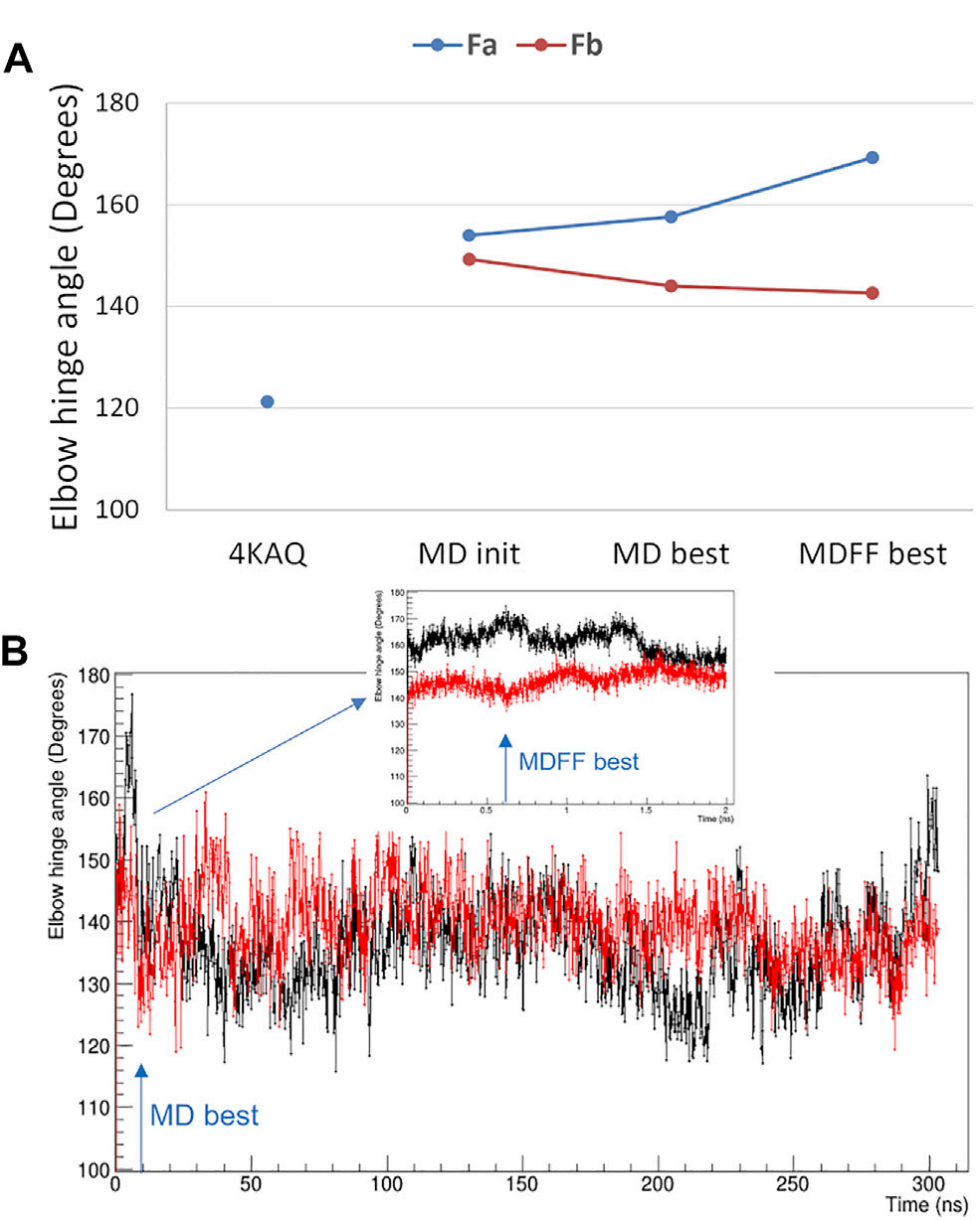
Elbow angle, i.e., the angle between the variable and constant Fab domains. **(A)** comparison of values measured from the crystal structure with PDB code 4KAQ and from both Fab domains of the full-length model used for MD simulations: initial model (MD init), best-fitting model in unrestrained MD (MD best) and best-fitting model in MDFF (MDFF best); **(B)** Fa (black line) and Fb (red line) elbow angle values as a function of the simulated time for the unrestrained MD and the MDFF simulation (inset). The simulation time corresponding to MD best and MDFF best is highlighted with arrows.

The elbow angle, which is the angle between the pseudo-two-fold axes defined by aligning the light chain portion of the variable and constant domains onto the heavy chain one, has been measured for both Fab domains of the present analysis. We observed that (Figure 5A): 1) the full-length values are much larger than that of the crystal structure (pdb code 4KAQ); *2*) the Fa and Fb values differ significantly, and the difference is enforced while applying the direct space SAXS restrain, i.e., in the best-fitting model obtained by the MDFF simulation. In addition, the analysis of the elbow angle values as a function of the simulation time (Figure 5B) reveals that the values of Fa fluctuates more than those of Fb.

The difference between the two Fab units is confirmed by the analysis of their C_α_ RMSD, which is 3.2 Å, larger than the difference of the individual units with the Fab crystal structure 4KAQ (2.9 Å and 2.6 Å for Fa and Fb, respectively).

### Modelling the Structural Flexibility

**FIGURE 6:**
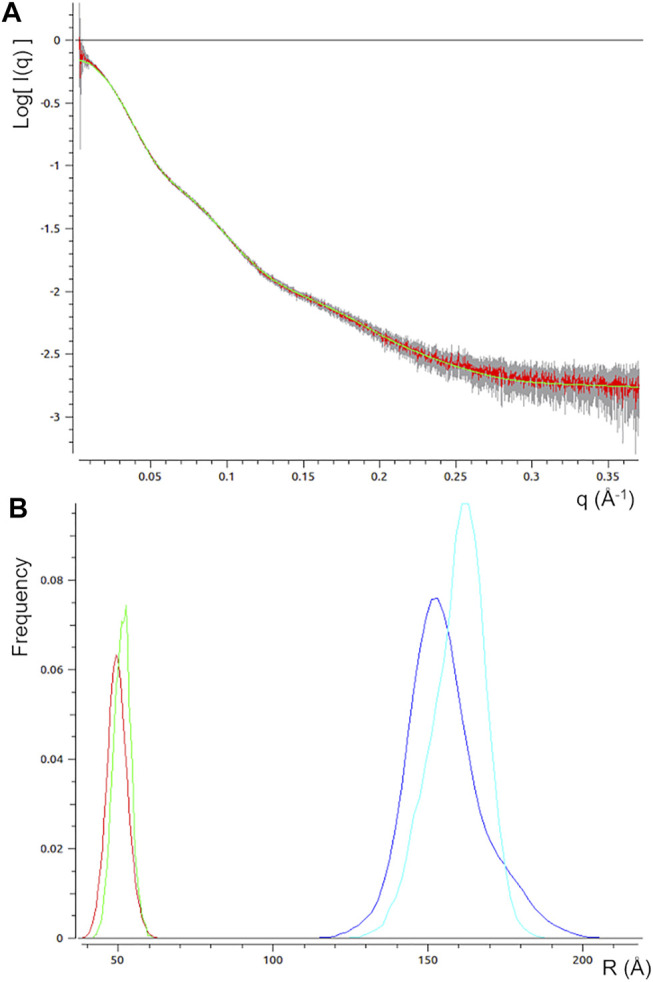
Results of the modelling procedure based on the ensemble optimization method. **(A)** best fit of the calculated SAXS profile (green line) vs the observed one (red line, experimental error bars in grey); **(B)** distribution of the radius of gyration (*R*_*g*_) and the maximum interatomic distance (*D*_max_) values for the selected models (green and cyan lines, respectively) and initial pool of random mAb structures (red and blue lines, respectively).

An alternative modelling approach has been followed, which is based on the ensemble optimization method implemented in the EOM program, where the intrinsic protein flexibility is accounted by considering the contemporary contribution of an ensemble of models. In this context, the time series analysis approach followed for static structural characterization, where individual models are generated and compared with experimental data, is replaced by a procedure where a set of models is generated according to the experimental *P(r)* and then combined to find the combination of models that best fit the SAXS profile. In this second approach, experimental restrains are still imposed in both direct and reciprocal space, but in a different way, more suited to account for the well-known mAb flexibility. Five models out of the 20,000 generated were selected as representative of the ensemble of mAb conformations after the EOM run. The SAXS profile calculated from these selected models fits very well the experimental one, with a χ^2^ = 0.33 (Figure 6A). The size distributions of the selected ensemble of models are shifted towards larger size with respect to the one of the initial random pool, and is still unimodal and symmetric (Figure 6B). This could be interpreted as due to a large number of almost similar conformations, none of which prevails over the others. The *R*_*flex*_ parameter, derived from the above size probability density functions by using the concept of information entropy, supplies a quantitative estimation of the flexibility of the system (Tria et al., 2015). For rituximab it has been estimated as 78.3% for the selected ensemble and 82.9% for the random pool, indicating a highly flexible system (*R*_*flex*_ = 100% for a fully flexible system, *R*_*flex*_ = 0% for a fully rigid system). Another metric for flexibility estimation is *R*_*sigma*_, defined as the ratio of the standard deviation of the selected ensemble and that of the random pool (Tria et al., 2015). *R*_*sigma*_ approaches 1.0 for a fully flexible system and it has been estimated as 0.82 for rituximab, confirming its high flexibility.

**FIGURE 7:**
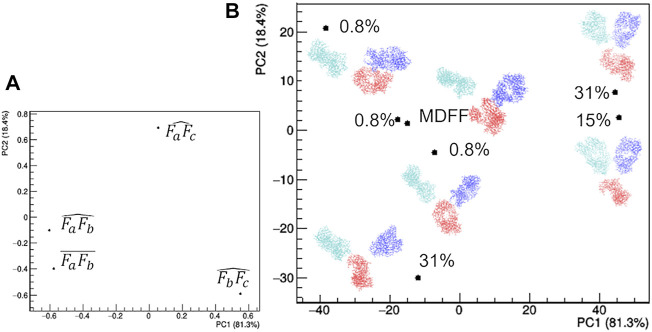
Comparison of the structural models generated by the ensemble optimization method based on principal component analysis (PCA) of related geometrical features. **(A)** PCA loadings values showing the individual geometrical parameters in discriminating the models; **(B)** PCA scores values showing the model discrimination. The models are drawn next to their representative points, with Fa, Fb and Fc coloured in cyan, blue and red, respectively, together with their relative weight in the EOM ensemble. The fraction of the total data variance explained by the first (PC1) and second (PC2) principal component is reported on relative the axes of the loadings (A) and scores (B) plots.

The selected models can be clearly distinguished in terms of their geometrical characteristics (Figure 7). The first principal component, which explains 81.3% of the total data variance, is dominated by the separation between the two Fab domains. In fact, both the distance between their two centers and the angle they form with the mAb center have large negative PC1 loadings (Figure 7A). The residual data variability (13.4%) is explained by the second principal component and is dominated by the orientation of the Fab with respect to the Fc. In fact, the angles formed by the center of mass of the Fab domains, the center of the antibody and the center of the Fc domain have large positive and negative PC2 loadings (Figure 7A). As a result, representative points of models having the largest weight within the EOM ensemble occupy the extremes of the scores plot, while two models having a weight of 0.8% assume an intermediate conformation and are placed at the center for the scores plot (Figure 7B). Asymmetric conformations, where Fa is separated from the rest of the antibody, are located at negative values of PC1 scores, as an effect of the separation introduced by the distance between Fa and Fb. It is worth noting that the model obtained by the static structural characterization through the MDFF-based procedure has negative PC1 values and it is placed between the two models with an intermediate conformation: it ideally represents an average of the EOM models. This result could be interpreted by considering that the large variability of mAb conformation is represented by a unimodal (large) distribution, thus it can be roughly approximated by a single conformation having intermediate structural features that falls around the mean of the distribution. Hence, as a first approximation the static MDFF model can be considered as representative of the average conformation of rituximab in solution, while the EOM models could be seen as representative of the largest conformational changes to which its domains are subjected.

### Study of Rituximab at Different Experimental Conditions

The comparative analysis of the conformations assumed by rituximab in solutions containing different buffers (A and B) or additives or put at different temperatures has been carried out at various levels: i) direct comparison of raw data by PCA; 2) comparison of geometrical parameters extracted from the SAXS signal (*R*_*g*_ and *D*_max_); 3) comparison of geometrical features of the atomistic models obtained by modelling the SAXS profiles by PCA; 4) comparison of the flexibility assessment as calculated by the EOM approach (*R*_*flex*_).

#### Dependence on Temperature

**FIGURE 8:**
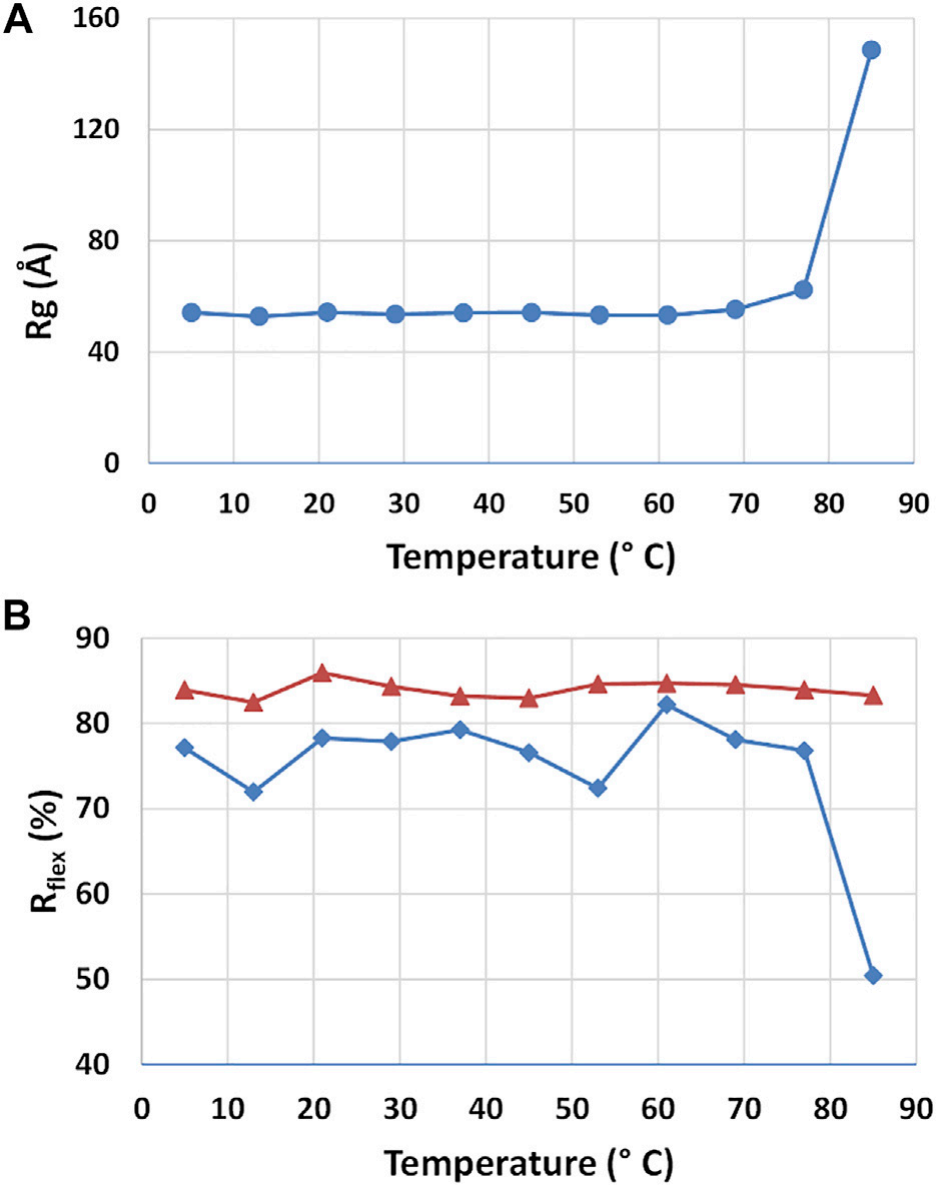
Results of the SAXS analysis of data taken at different temperatures. Radius of gyration *R*_*g*_ **(A)** and *R*_*flex*_ parameter determined by the ensemble optimization method for the selected structures (blue line) and the pool of generated structures (red line) **(A)**.

The stability of the rituximab structure and the structural changes induced by temperature was studied by using SAXS datasets taken at different storage temperature. *P(r)* calculations and EOM flexibility analysis was performed on each dataset independently, allowing to determine the radius of gyration and the *R*_*flex*_ parameter as a function of temperature (Figure 8). The radius of gyration of the antibody is stable up to 60°C and moderately increase from 60° to 80°C. After 80°C, it significantly increases, suggesting a complete unfolds of the protein. Such temperatures agree very well with those shown in the literature for the thermal denaturation of this antibody (Andersen et al., 2010), where it is reported that C_H_2 of the Fc region unfold at 69°C, the Fab domain at 75°C and C_H_3 of the Fc region at ∼83°C. Unexpectedly, the EOM analysis does not reveal any systematic change in flexibility (*R*_*flex*_) up to 60°C, similarly to *R*_*g*_. For higher temperature *R*_*flex*_ undergoes a significant decrease, a result that can be interpreted as a reduction of the population size due to the increasing weight of unfolded configurations.

#### Dependence on mAb Concentration

**FIGURE 9:**
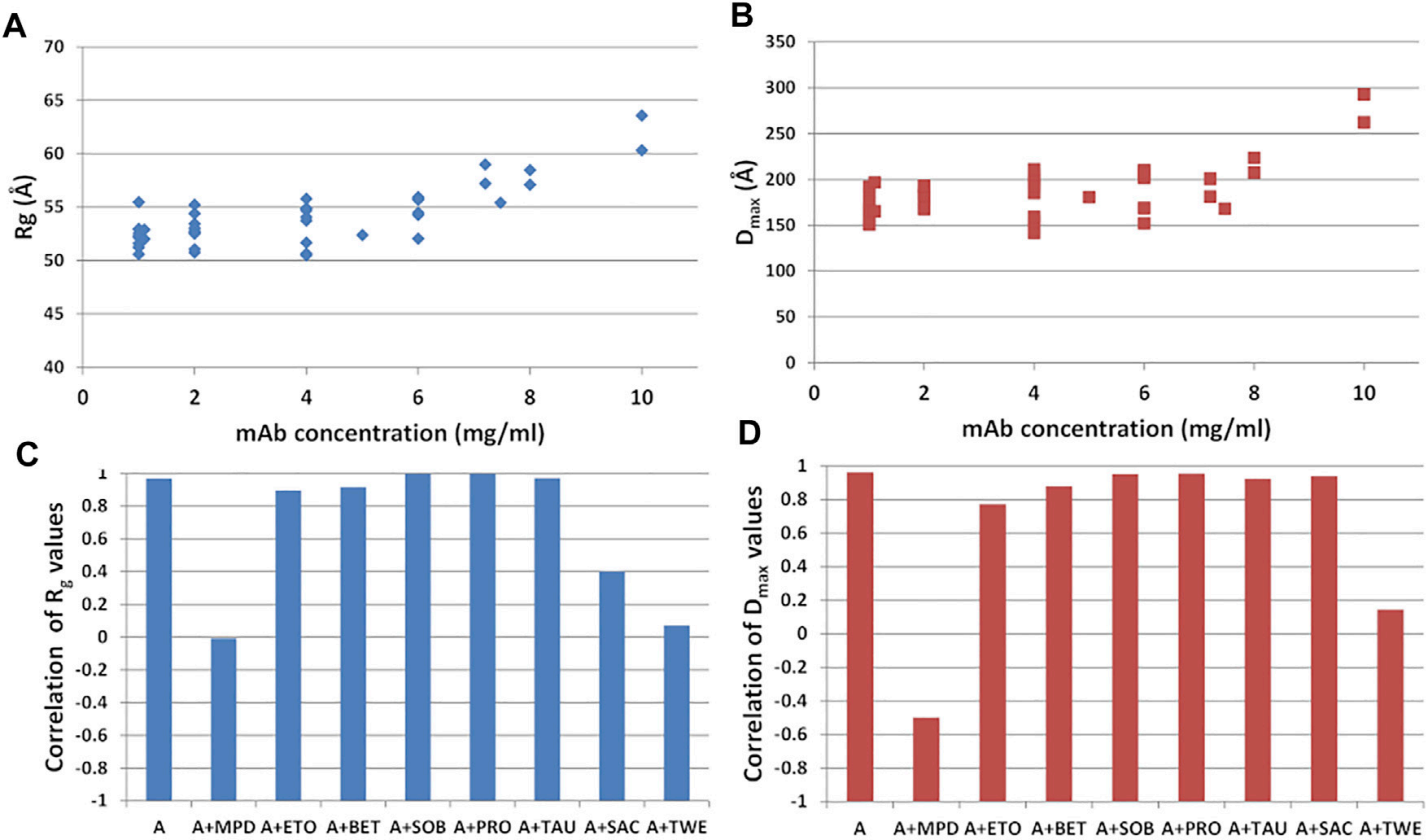
**(A)** Radius of gyration (*R*_*g*_) and **(B)** maximum inter-particle distance (*D*_max_) calculated from SAXS data as a function of the mAb concentration. Pearson’s correlation factor for *R*_*g*_ **(C)** and *D*_max_ **(D)** against concentration values. Samples refer to mAb in buffer A and in the presence of the additives listed in Supplementary Table S1.

Different mAb concentrations were tested during the SAXS experiments to optimize the signal-to-noise ratio for the X-ray scattering intensity. Moreover, the effect of the additives listed in Supplementary Table S1, namely sucrose (SAC), sorbitol (SOB), ethanol (ETO) (+/-)-2-Methyl-2,4-pentanediol (MPD), polysorbate 80 (TWE), proline (PRO), betaine (BET), taurine (TAU), was monitored, to search for peculiar interactions modulating the static mAb structure or its flexibility properties. They include a selection of the most used polyalcohol (SAC, SOB), kosmotropic (BET, PRO, TAU), surfactant (TWE), organic nonvolatile (MPD) and volatile (EtOH) additives in protein crystallization showing an effect towards protein stability. This bunch of data allowed us to study the shape of the antibody as a function of its concentration in solution. Results, shown in Figures 9A,B, indicate that no significant change in the structural parameters are observed as a function of concentration in the range from 1 to 4 mg/ml, in agreement with previous observations (Mayans et al., 1995; Boehm et al., 1999; Furtado et al., 2004). However, a simultaneous increase of *R*_*g*_ and *D*_max_ is observed for concentrations above 4 mg/ml, which could be due to aggregation effects. Interestingly, aggregation effects can be modulated by the presence of specific additives. In fact, the general trend of increase in size of the protein as concentration increases, which can be monitored by the Pearson’s correlation factor for *R*_*g*_ or *D*_max_ against concentration values, is not followed in the case of solutions of rituximab added with SAC, TWE and MPD (Figure 9C). Correlation values close to 0 are obtained for TWE and MPD, consistently for *R*_*g*_ and *D*_max_, while SAC shows a decrease of correlation only in the case of *R*_*g*_ *.*

#### Effect of Additives

**FIGURE 10:**
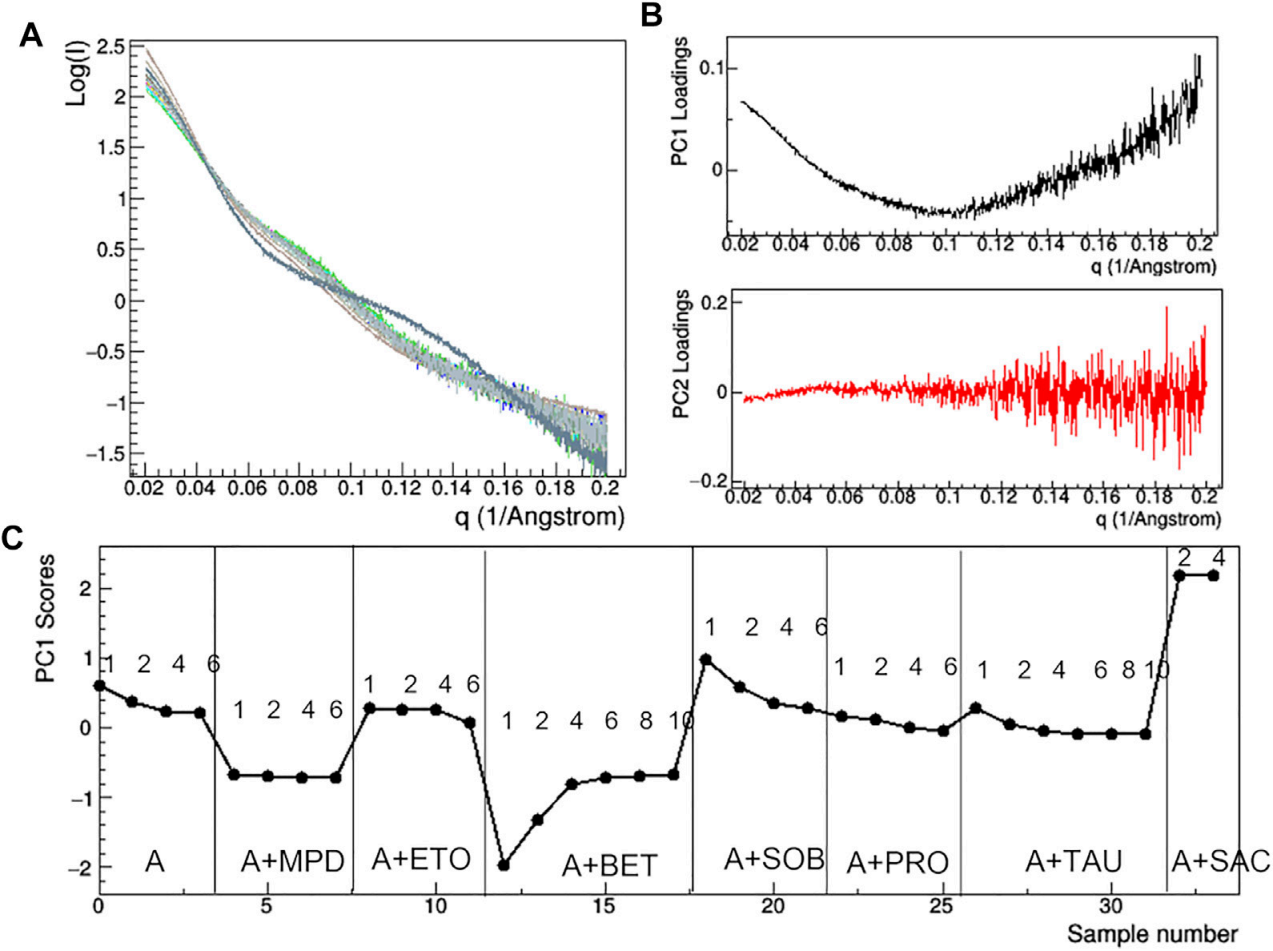
PCA analysis of the raw SAXS data taken for rituximab in solutions containing different additives. Superposition of the individual SAXS profiles, representing the data matrix supplied to PCA **(A)**; loadings of the first (PC1) and second (PC2) principal components **(B)**; PC1 scores as a function of the dataset considered, with mAb concentration in mg/ml and type of additive reported **(C)**. Plots B and C have been obtained after removing the profile of the sample A + TWE from the data matrix, as it produces large deviations in the PCA.

At the lowest mAb concentrations (1–2 mg/ml), aggregation effects are negligible and the size of the protein still ensures a sufficient signal-to-noise ratio to conduct a comparative analysis on the effect of additive. Comparison of raw SAXS profiles has been conducted by PCA (Figure 10). The loading plots (Figure 10B) suggest that the first principal component (PC1) captures changes in slope of the SAXS curve, while the second one (PC2) is sensitive to the fluctuation of the SAXS signal at high *q*, so that PC2 is not relevant for the comparative analysis. The PC1 scores (Figure 10C) show anomalies introduced by the presence of TWE, SAC, ABET and MPD: the first one produces a highly different SAXS profile, which has been excluded by PCA, while the other additives produce large deviations from 0 of the PC1 scores. In addition, the PCA confirms the absence of concentration effects for MPD and SAC, while highlighting large dependence on concentration in the presence of BET and SOB.

**FIGURE 11:**
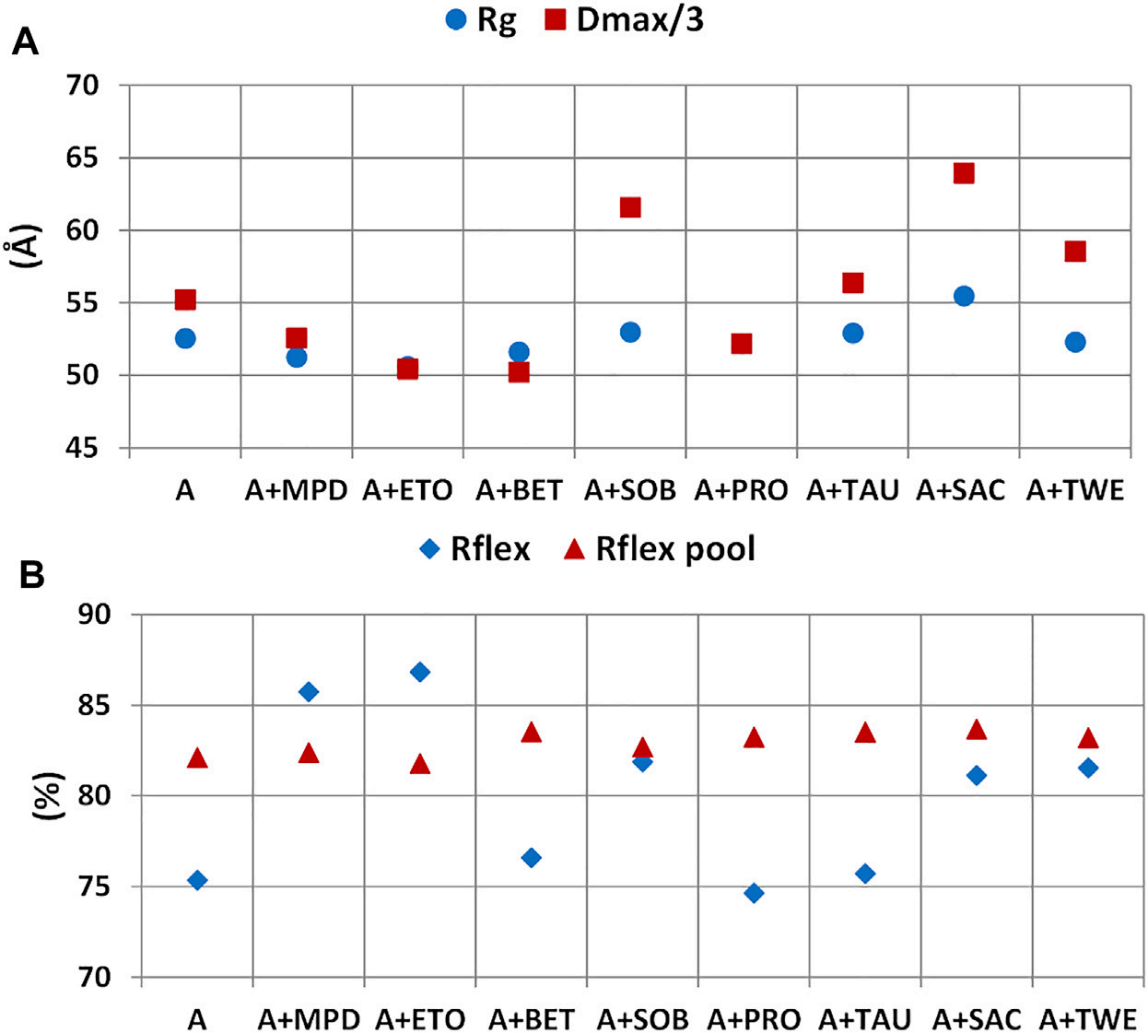
Effect of additives on structural parameters of rituximab derived from SAXS data. Radius of gyration (*R*_*g*_) and maximum inter-particle distance (*D*_max_) **(A)** and *R*_*flex*_ figure of merit for the generated pool of structures and those selected **(B)**.

At the level of rituximab structural properties derived by SAXS, the static size parameters *R*_*g*_ and *D*_max_ and the flexibility figure of merit *R*_*flex*_ calculated for the random pool of generated structures and those selected by the EOM procedure have been considered (Figure 11). It can be noted a significant increase in the size of the scattering object in the presence of SAC with respect to the case were no additives were added (A). Among the other additive tested, SOB and TWE give a higher inter-particle distance than A, while ETO and BET give a lower one. It is interesting to note the similar trend of the *R*_*g*_ */D*_max_ in Figure 11A with the PC1 scores shown in Figure 10C: upward deviations of *R*_*g*_ */D*_max_ respect to those calculated in the absence of additives correspond to positive PC1 scores (SAC, SOB), whilst downward deviations correspond to negative PC1 scores (BET, MPD). The flexibility of the selected pool is increased by most of the additives, apart from BET, PRO, and TAU. For MPD and ETO *R*_*flex*_ is even higher that of the pool of generated structures. Instead the flexibility the pool of generated structure is not affected by the presence of additives, as the generation is driven by the sequence of the antibody.

**FIGURE 12:**
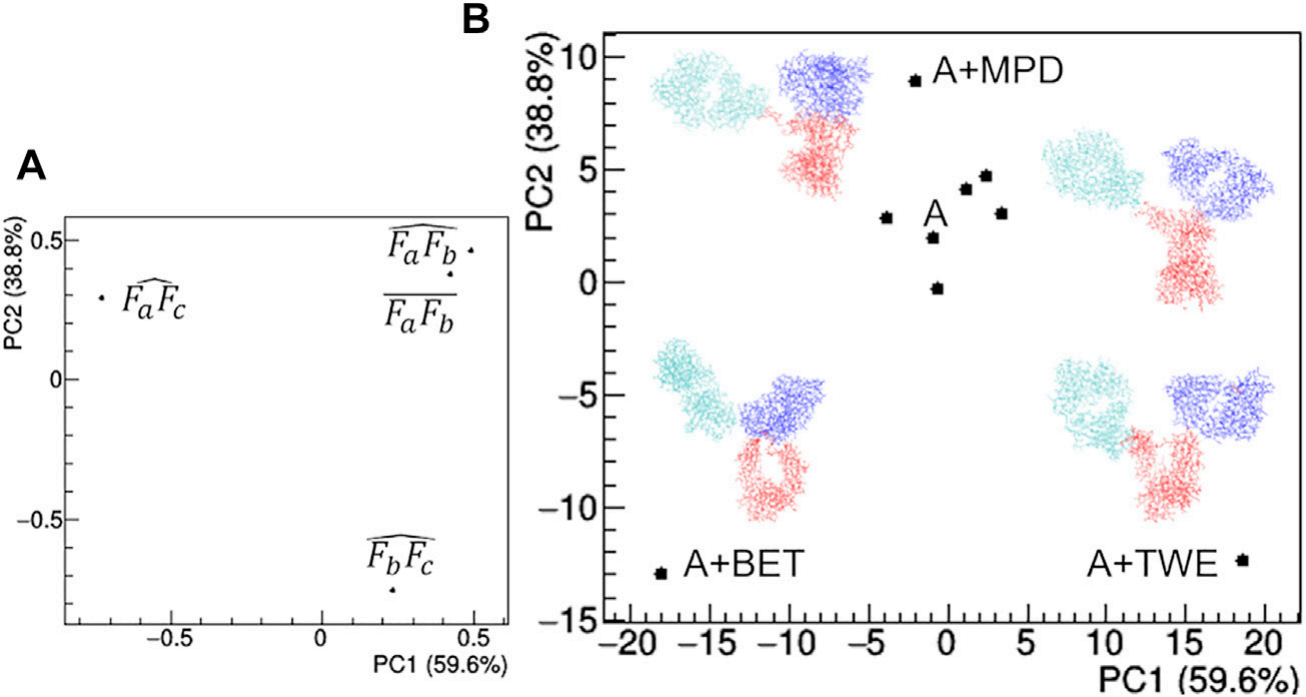
Comparison based on PCA of geometrical features of rituximab models obtained from SAXS measurements in the presence of several additives. **(A)** PCA loadings values showing the role of individual geometrical parameters in discriminating the models; **(B)** PCA scores values showing the model discrimination. The models are drawn next to their representative points, with Fa, Fb and Fc coloured in cyan, blue and red, respectively. The fraction of the total data variance explained by the first (PC1) and second (PC2) principal component is reported on relative the axes of the loadings (A) and scores (B) plots.

The comparison of structural models derived by SAXS data (Figure 12) highlights large deviations occurring in the presence of TWE, BET and MPD additives, while the remaining additives do not produce relevant deviations with respect to the model obtained in the absence of additives. The main geometrical parameters discriminating the models are: *i*) the highest distance between Fa and Fc for TWE, *2*) the highest angle between Fa and Fc for BET and the highest angle between Fb and Fc for MPD. It is worth noting that SAC does not produce a significant modification of the protein model with respect to that obtained in the absence of additives.

#### Interactions With Sugars

**FIGURE 13:**
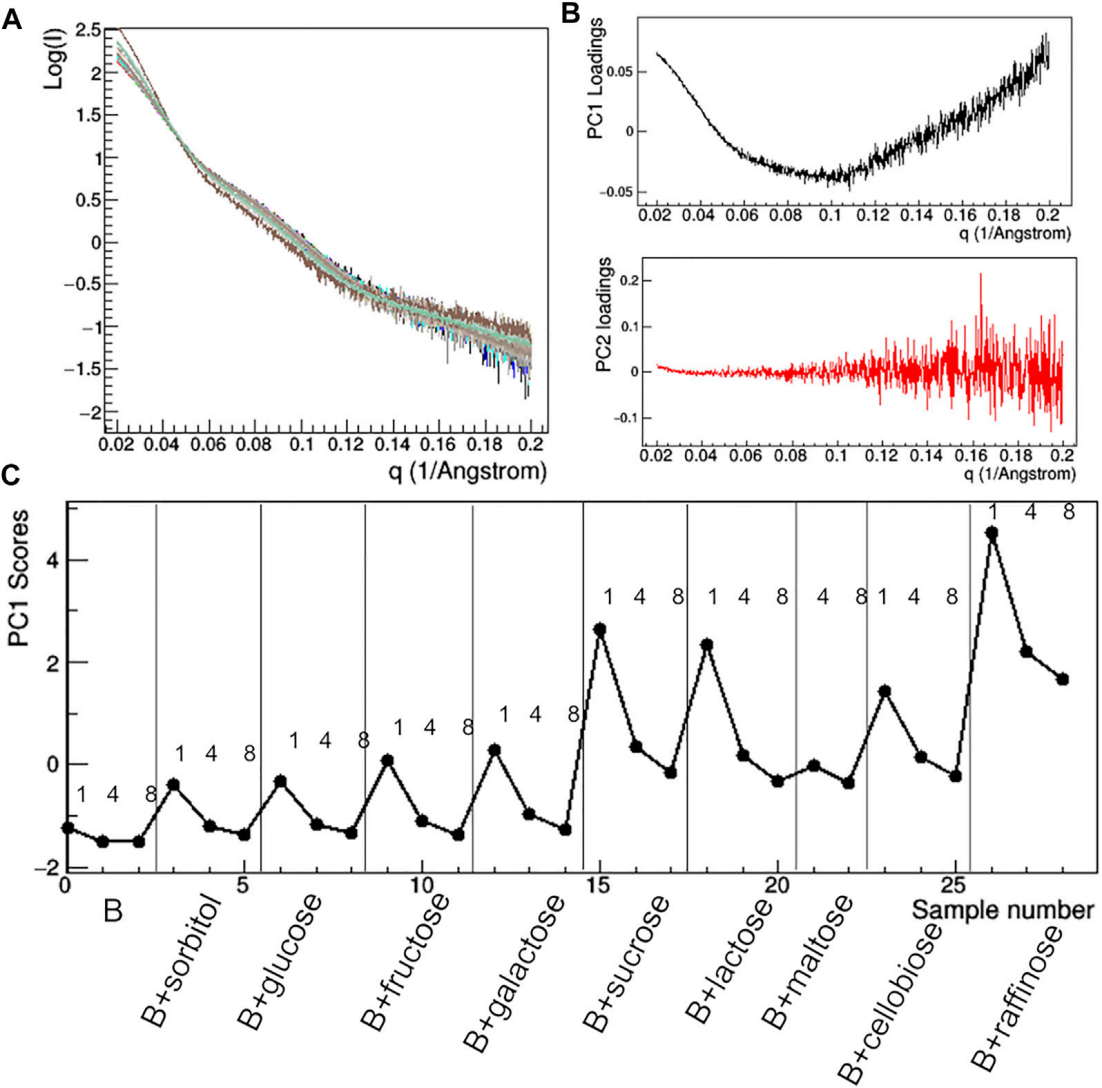
PCA analysis of the raw SAXS data taken for rituximab in solutions containing different sugars. Superposition of the individual SAXS profiles, representing the data matrix supplied to PCA **(A)**; loadings of the first (PC1) and second (PC2) principal components **(B)**; PC1 scores as a function of the dataset considered, with mAb concentration in mg/ml and type of additive reported **(C)**.

**FIGURE 14:**
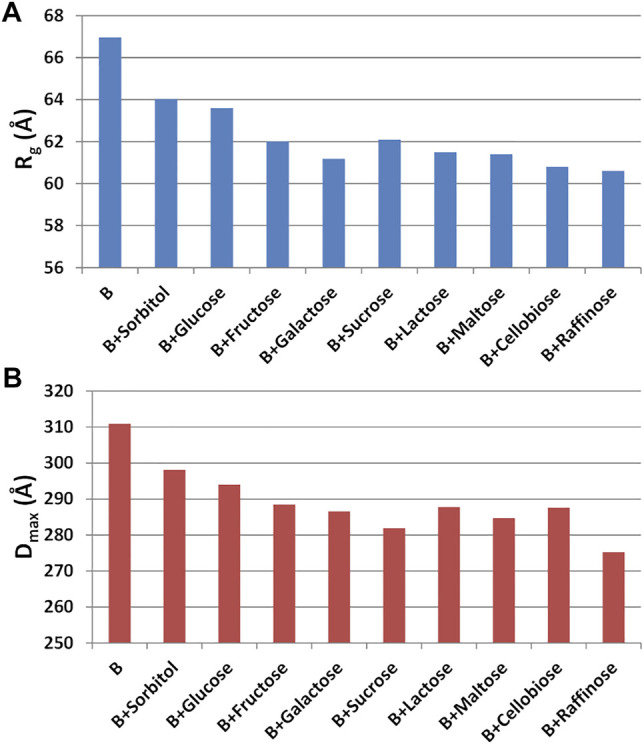
**(A)** Radius of gyration (*R*_*g*_) and **(B)** maximum inter-particle distance (*D*_max_) measured for rituximab in buffer B at 8 mg/ml and in presence of mono-, di- and tri-saccharides.

Having found an anomalous behavior of the SAXS signal when sucrose (SAC) is used as additive, a systematic study about the effect of several saccharides on the mAb was launched. Mono-, di-, and tri-saccharides (Supplementary Table S2) were added to rituximab in buffer B and solutions were monitored by SAXS measurements. The comparative analysis of raw data (Figure 13) shows that PC1 scores are able to capture the changes in the slope of the SAXS radial profile, similarly to what found for the study with additives (Figure 10). PC1 scores indicate an increasing effect on the shape of the scattering curve as the molecular weight of the sugar increases, an effect which holds at all the three concentrations monitored (1, 4, and 8 mg/ml). When considering the size parameters extracted from the SAXS data, the same trend can be found (Figure 14), with *R*_*g*_ and *D*_max_ that systematically reduce as the molecular weight of the sugar increases. However, these features only hold at 8 mg/ml, while at lower concentration no relevant dependence of the size parameters on the sugar added was found (data not shown).

**FIGURE 15:**
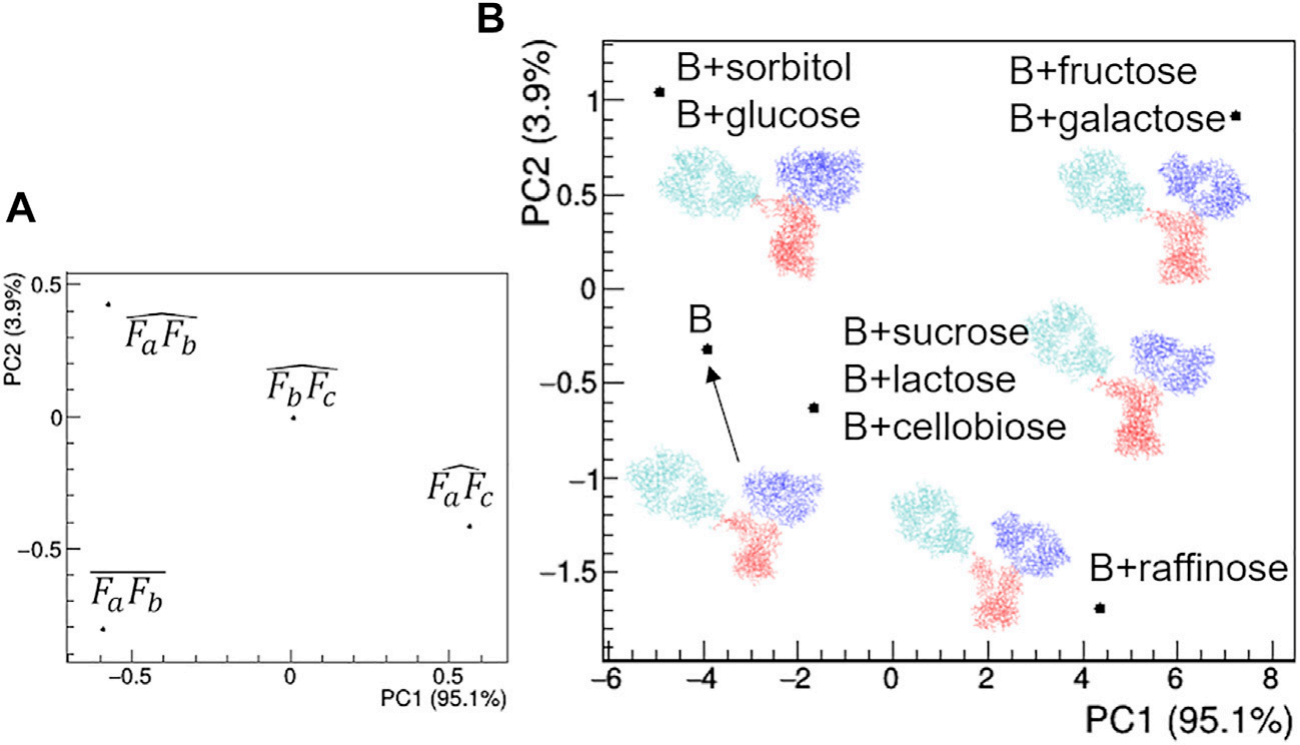
Comparison of the structural models based on PCA of geometrical features of rituximab models obtained from SAXS measurements in the presence of several additives. **(A)** PCA loadings values showing the role of individual geometrical parameters in discriminating the models; **(B)** PCA scores values showing the model discrimination. The models are drawn next to their representative points, with Fa, Fb and Fc coloured in cyan, blue and red, respectively. The fraction of the total data variance explained by the first (PC1) and second (PC2) principal component is reported on relative the axes of the loadings (A) and scores (B) plots.

The comparative analysis of the modelling results (Figure 15) shows that the rituximab structural model is less perturbed by saccharides than by other additives, confirming the result obtained by comparing the effect of SAC in Figure 12. In particular, the relative positioning of the two Fab arms and the Fc domain is only slightly affected by the interaction with sugars. The above-mentioned mono- < di- < tri-saccharides hierarchy is established along the PC2 direction (Figures 15B,C), which is dominated by changes in the mutual orientation of Fa and Fb domains (Figures 15A,C). In addition, it can be noted that the structural model obtained for buffer B is similar to that obtained for buffer A, and the same applies for the *R*_*g*_ values at the same protein concentration, which rules out major structural changes of rituximab triggered by pH variations (pH increases from 6.5 to 8.0 going from A to B).

## Discussion

Acquiring structural knowledge is fundamental to deeply understand the mechanism of action of rituximab and possibly improve it. Many new findings have been raised since now by using different techniques. Individual rituximab domains Fc (Tang and Chen, 2016) and Fab (Bzymek and Williams, 2014), as well as the interaction between Fab and the epitope of the CD20 protein (Du et al., 2007 and Du et al., 2008), have been extensively investigated by X-ray diffraction. Recently cryo-EM has been used to disclose the structure of full-length CD20 in complex with the rituximab Fab domain (Rougè et al., 2020). However, the structure of the full-length antibody is still lacking, due to its inherent flexibility, which has frustrated all attempts made so far to produce well-diffracting crystals (Yang et al., 2019) and hindered the use of cryo-EM due to the problematic clustering of cryo-EM images. In this context, SAXS offers the unique chance to characterize the low-resolution structure of the full-length rituximab, with the additional advantage to infer information about its behavior in solution.

The pair distribution function and the *ab initio* modelling of SAXS data displayed an asymmetric disposition of the Fab arms relative to the Fc portion, a conformation that has been found also for other human IgG1 antibodies (Ashish et al., 2010; Tian et al., 2014). It is worth noting that the presence of two peaks in the *P(r)* curve is not a common feature of antibodies. In fact, a single peak was found when analyzing SAXS/SANS data from bovine IgG (Boehm et al., 1999), which was interpreted as due to a high level of flexibility across the hinges that allows the Fab arms to adopt a continuous range of conformations relative to Fc. Therefore, the bimodal shape of *P(r)* points to a reduced flexibility across the hinges, with a consequent preferential asymmetry in the placement of the two Fab arms with respect to the Fc portion.

The radius of gyration and maximum particle dimension have been derived for rituximab in different buffers, at different temperatures and in the presence of additives. The exact knowledge of these structural parameters is of paramount importance to simulate the crystallization behavior of the antibody in the framework of theoretical models for diffusion-limited nucleation of macromolecules in solution (Lutsko, 2019). The *R*_*g*_ value of rituximab at pH 6.5 is compatible with that recently reported by Narvekar et al. (2020).

In addition, SAXS data in reciprocal and direct space have been here used to restrain the atomistic modelling of the full-length antibody in the framework of two protocols based on completely different assertions. A full characterization of the individual atomic positions has been attempted by complementing the limited SAXS data resolution with MD simulations carried out on an initial structure derived by homology modelling. Such approach takes advantage of a preliminary unrestrained exploration of the phase space, followed by a local exploration restrained by experimental data in direct space (MDFF). The reciprocal space has then been used to identify the simulated model whose calculated scattering best agrees with SAXS data. The advantage of this strategy is that it supplies information about the average intra-domain configuration, since the whole protein model is treated as fully flexible. In our study this approach has been used to measure the elbow angle, which has been recently related to the dynamics of antigen-binding fragment (Fernández-Quintero et al., 2020). The disadvantage is that the system is described by using a unique conformation, which is not particularly suited for a flexible system. On the other hand, a second approach has been followed, where the flexibility of the system is accounted for by selecting an ensemble of models best describing the SAXS data, out of a large number of generated models. This approach has the advantage of being more effective in modelling the intrinsic mAb flexibility, but has the disadvantage of describing the each domain of the antibody (Fa, Fb and Fc) as rigid unit. It is thus only suited to characterize the most probable inter-domain configurations.

Despite the limited resolution of SAXS data interesting structural determinants has been revealed by combining experimental data with computational modelling. The elbow angle measured from the MDFF model perfectly fits the expected distribution of IgG1 antibody, as determined by MD simulations (Fernández-Quintero et al., 2020), but it is larger than the experimental values derived by the X-ray crystal structure of the rituximab Fab (Bzymek and Williams, 2014). This finding is relevant since the elbow angle has been related to Fab flexibility and to the ability of the antibody to recognize different antigens (Landolfi et al., 2001; Stanfield et al., 2006; Niederfellner et al., 2011). Moreover, the larger fluctuation of the elbow angle values for Fa with respect to those of Fb can be linked to the larger distance of the Fa domain from the center of the antibody, so that a correspondence between asymmetry in Fab placing and elbow angle dynamics could be conceived.

The dependence on temperature highlighted an interesting connection between geometric parameters of the average structure and the composition of the ensemble of representative structures determined by the EOM approach. Both features remain constant up to 60°C, undergo a moderate change from 60°C to 80°C and then diverge above 80°C. In addition, the increase of structural size due to the mAb unfolding is strictly related to a decrease in the number of possible protein configurations, thus a reduction of its conformational flexibility. The reduction of conformational entropy of the partially unfolded state of the mAb should be related to the loss of degrees of freedoms of its original domains. It is worth noting that a recent study carried out on a IgG1 antibody by using differential scanning calorimetry (Garidel et al., 2020) showed a pre-transition at 54°C and a main transition at 75°C in the molar heat capacity. Our results do not evidence any pre-transition, and attest a higher unfolding transition.

The study of solution scattering in the presence of additives highlighted their role in modulating the structural properties of rituximab. Aggregation effects arising at high mAb concentration (above 4 mg/ml) can be reduced by adding sucrose, or completely cancelled by adding surfactants, such as MPD or TWE. This may probably be due to the fact that these compounds are able to modulate the mAb surface properties, thus reducing the inter-molecular interactions that normally take place in highly concentrated solutions. The analysis of the structural properties of mAb at lower concentrations reveals that the molecular mechanism responsible for this anti-aggregation effect is indeed different for the three additives: while SAC produces a significant increase in the *R*_*g*_ and *D*_max_ of the protein structure, thus its binding affects SAXS data, TWE only produces an increase in *D*_max_, and MPD determines a slight decrease in both parameters. Thus surfactants (MPD and TWE) do not stably bind the antibody on specific sites, rather they interact with its surface with weaker polar interactions. Surfactants are also able to increase the mAb flexibility, probably hindering the normal inter-domain interactions that occurs in the antibody. This property is also shared by other organic additives, such as ETO, SOB and SAC, while BET, PRO and TAU do not affect the protein flexibility. Among the various additive tested, BET showed relevant properties, as it is able to reduce the mAb size (Figure 11) as due to the shortening of the distance between the two Fab arms (Figure 12), and to highly modulate these properties as a function of the protein concentration (Figure 10).

The main effect of the presence of saccharide additives on the structural properties of rituximab is a systematic decrease of the size parameters as a function of the sugar complexity at high antibody concentrations (8 mg/ml). In particular, in these conditions the shape of the SAXS profile was found to follow a mono- < di- < tri-hierarchy. The direct effect of saccharides on the modelling of antibody is less evident than that of surfactants or betaine, and results in slight changes of the mutual placement of the mAb domains, mainly due to their different orientation. The presence of sugar molecules possibly bound on the antibody surface could be responsible for altered inter-domain interactions, but unfortunately the resolution of SAXS data does not allow the modelling of the sugar interaction at atomic details.

The collected evidence is of importance to found possibly additives for rituximab crystallization, which represents a mean of purification at industrial level alternative to column A chromatography (http://www.amecrys-project.eu).

## Conclusion

The structural determinants of the rituximab in solution have been disclosed by using SAXS, one of the most thorough techniques to investigate the protein structure also in the presence of high flexibility of the system. The limited SAXS data resolution has been compensated to intensive use of computational modelling techniques, which embed *a priori* knowledge on stereochemistry and allows to introduce restraints based on experimental data in both reciprocal and direct space. Such methodological effort has allowed to reveal new findings related to the rituximab structure, such as the unexpected structural difference between the two arms of the Fab domain, which could be related to the asymmetry of their spatial arrangement with respect to the Fc domain. An increased elbow angle fluctuation in the more distant Fab unit has been also found. In addition, the conformational flexibility of rituximab in solution has been assessed, confirming that it is due to small fluctuations of the relative position of its domain. In particular, the distance between the two arms of the Fab domain and the angles they form with Fc are the relevant geometric variables determining the dynamics of the antibody.

The static structural parameters and the amount of structural flexibility of rituximab has been studied as a function of temperature, protein concentration in solution, and the presence of additives. Surfactants were found to reduce aggregation effects occurring in solution at high protein concentration, while sucrose was identified as relevant additive to reduce aggregation and increase the antibody average size, which points to a probably stable interaction of sugar molecules with exposed sites. Betaine is instead able to reduce the mAb average dimensions. A systematic reduction of the antibody average size at high concentration was achieved by using saccharides as additives, with an amount scaling with sugar complexity.

Altogether, these results reveal new hints about the conformational behavior of rituximab in solution and a deeper understanding of its structural properties, disclosing the possibility of a more effective design of next-generation anti-CD20 mAbs. In fact, understanding how the relative interdomain orientations and the elbow angle influence antigen specificity, affinity, and stability has broad implications in the field of antibody modeling and engineering. In addition, a careful analysis of the effect of additives on the mAb structure, and in particular of their influence on inter-domain and inter-particle interactions, is of paramount importance to find new routes for mAb crystallization, a method that could replace in the future the classical purification step by column A chromatography. From the methodological point of view, the study provides a new combined experimental/computational workflow to be used for the structural characterization of highly flexible proteins, where the SAXS data are modelled by using the MDFF tool.

## Data Availability

The datasets presented in this study can be found in online repositories. The names of the repository/repositories and accession number(s) can be found below: https://www.sasbdb.org/data/SASDMX3.
