# Supplementary material for: Structural Characterization of the Full-Length Anti-CD20 Antibody Rituximab
Source: Front Mol Biosci. 2022 Apr 11;9:823174. doi: 10.3389/fmolb.2022.823174 (PMC9037831; doi:10.3389/fmolb.2022.823174)
Supplement: Supplementary file 1 [file Presentation1.pdf]

Supplementary Material

Structural characterization of the full-length anti-CD20 antibody rituximab

Benny Danilo Belviso[1], Giuseppe Felice Mangiatordi[1], Domenico Alberga[2], Vincenzo Mangini[1], Benedetta Carrozzini[1], Rocco Caliandro[1]*

[1]Institute of Crystallography, CNR, Bari, Italy

[2]Cineca, Casalecchio di Reno, Italy

*** Correspondence:**
Corresponding Author: rocco.caliandro@ic.cnr.it

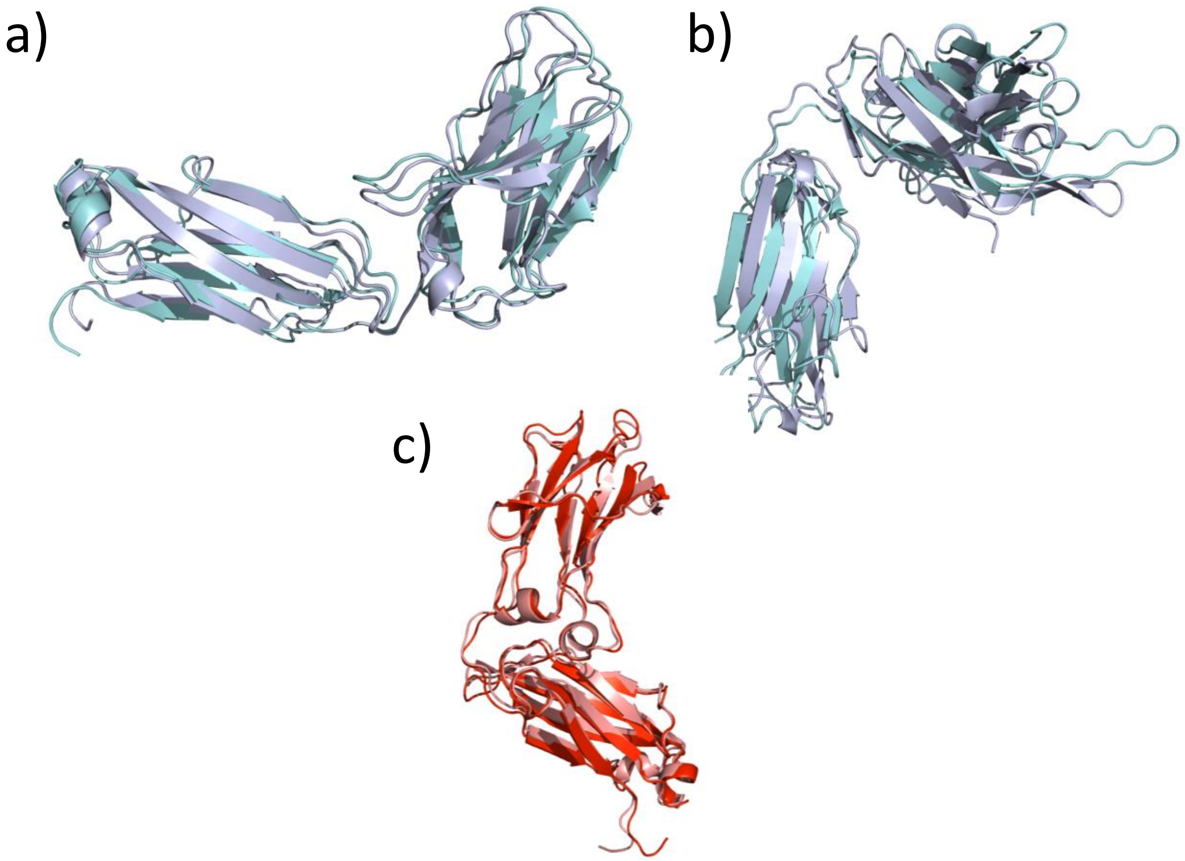

Figure S1. Superposition between the machine learning (lilac and light red) and the homology modelling (cyan and dark red) predictions of the light chain (a), Fab portion heavy chain (b) and Fc portion heavy chain (c).

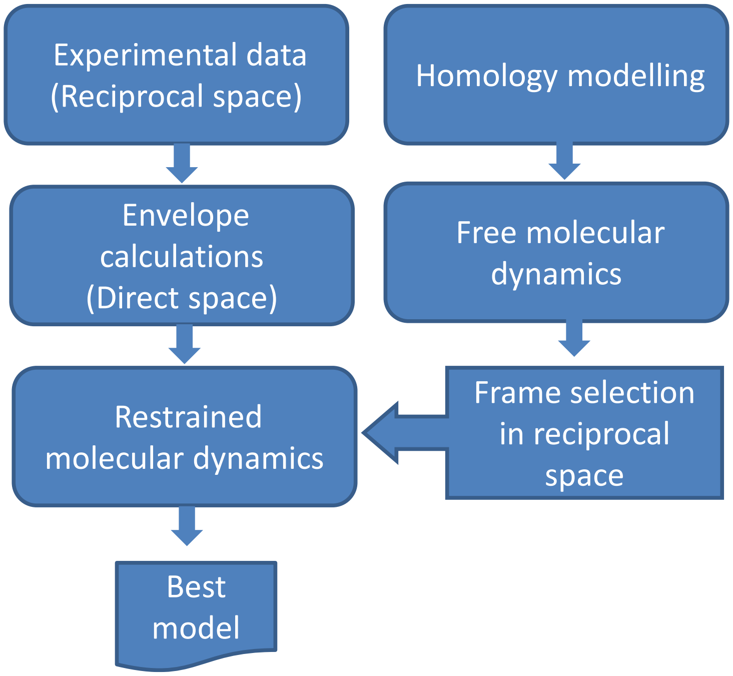

Figure S2. Scheme of the experimental data analysis integrated with computational modelling. Best model is obtained from SAXS data as a result of a procedure that uses experimental information in reciprocal (SAXS radial profiles) and direct (SAXS envelopes) space.

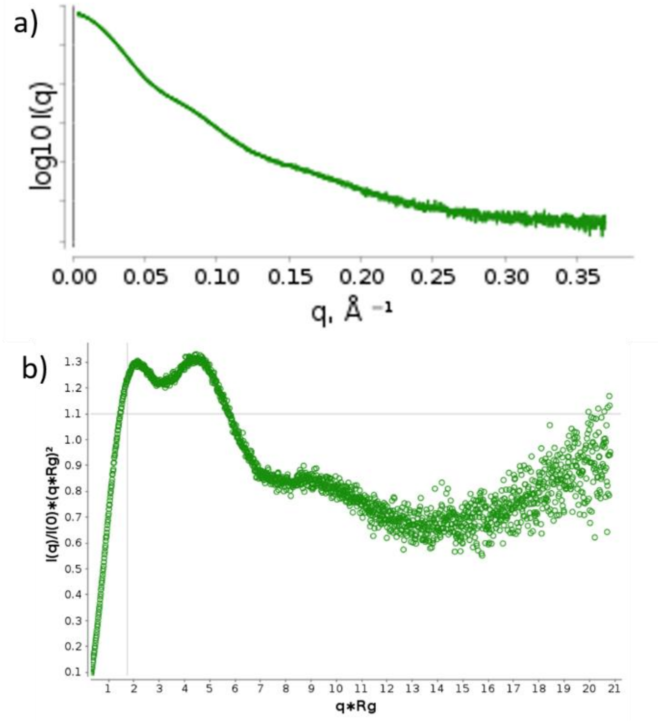

Figure S3. Logarithmic profile (a) and normalized Kratky plot (b) from SAXS measurements on rituximab. The position of the peak expected for compact globular scatters is shown by thin horizontal and vertical lines.

Table S1. Additives tested in solutions containing Rituximab.

Code	Extended name	IUPAC name	Properties
**MPD**	MPD	2-Methylpentane-2,4-diol	Surfactant and emulsion-stabilizer
**ETO**	EtOH	Ethanol	Alcohol naturally produced by the fermentation of sugars
**BET**	Betaine	(Trimethylazaniumyl)acetate	Amino acid derivative occurring in plants
**SOB**	Sorbitol	(2S,3R,4R,5R)-Hexane-1,2,3,4,5,6-hexol	Sugar alcohol
**PRO**	Proline	Pyrrolidine-2-carboxylic acid	Proteinogenic amino acid
**TAU**	Taurine	2-Aminoethane-1-sulfonic acid	Antioxidant, osmoregulator, membrane stabilizer
**SAC**	Saccharose	β-D-Fructofuranosyl α-D-glucopyranoside	Disaccharide
**TWE**	Polysorbate 80	Polyoxyethylene (20) sorbitan monooleate	Non-ionic surfactant and emulsifier

Table S2. Sugars of different complexity tested in solutions containing Rituximab.

**Name**	**Type**	**Chemical formula**	**Chemical structure**
Sorbitol	Monosaccharides	$C_6H_{14}O_6$	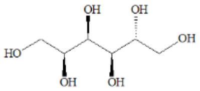
Glucose	Monosaccharides	$C_6H_{12}O_6$	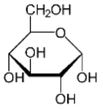
Fructose	Monosaccharides	$C_6H_{12}O_6$	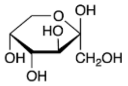
Galactose	Monosaccharides	$C_6H_{12}O_6$	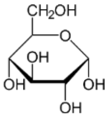
Sucrose	Disaccharides	$C_{12}H_{22}O_{11}$	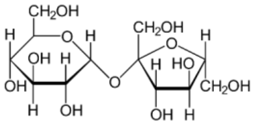
Lactose	Disaccharides	$C_{12}H_{22}O_{11}$	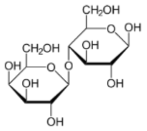
Maltose	Disaccharides	$C_{12}H_{22}O_{11}$	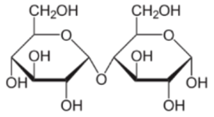
Cellobiose	Disaccharides	$C_{12}H_{22}O_{11}$	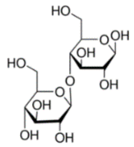
Raffinose	Trisaccharides	$C_{18}H_{32}O_{16}$	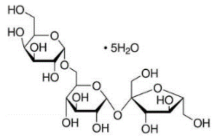
